# Microphysiologic Human Tissue Constructs Reproduce Autologous Age-Specific BCG and HBV Primary Immunization *in vitro*

**DOI:** 10.3389/fimmu.2018.02634

**Published:** 2018-11-20

**Authors:** Guzman Sanchez-Schmitz, Chad R. Stevens, Ian A. Bettencourt, Peter J. Flynn, Klaus Schmitz-Abe, Gil Metser, David Hamm, Kristoffer J. Jensen, Christine Benn, Ofer Levy

**Affiliations:** ^1^Division of Infectious Diseases, Boston Children's Hospital, Boston, MA, United States; ^2^Precision Vaccines Program, Boston Children's Hospital, Boston, MA, United States; ^3^Harvard Medical School, Harvard University, Boston, MA, United States; ^4^Division of Newborn Medicine, Boston Children's Hospital, Boston, MA, United States; ^5^Broad Institute of Harvard and MIT, Cambridge, MA, United States; ^6^Adaptive Biotechnologies, Seattle, WA, United States; ^7^Research Center for Vitamins and Vaccines, Bandim Health Project, Statens Serum Institut, Copenhagen, Denmark; ^8^Bandim Health Project, Indepth Network, Bissau, Guinea-Bissau; ^9^Department of Biotechnology and Biomedicine, Technical University of Denmark, Kgs Lyngby, Denmark

**Keywords:** microphysiology, model, newborn, vaccine, immunization, autologous, tissue engineering

## Abstract

Current vaccine development disregards human immune ontogeny, relying on animal models to select vaccine candidates targeting human infants, who are at greatest risk of infection worldwide, and receive the largest number of vaccines. To help accelerate and de-risk development of early-life effective immunization, we engineered a human age-specific microphysiologic vascular-interstitial interphase, suitable for pre-clinical modeling of distinct age-targeted immunity *in vitro*. Our Tissue Constructs (TCs) enable autonomous extravasation of monocytes that undergo rapid self-directed differentiation into migratory Dendritic Cells (DCs) in response to adjuvants and licensed vaccines such as Bacille Calmette-Guérin (BCG) or Hepatitis B virus Vaccine (HBV). TCs contain a confluent human endothelium grown atop a tri-dimensional human extracellular matrix substrate, employ human age-specific monocytes and autologous non heat-treated plasma, and avoid the use of xenogenic materials and exogenous cytokines. Vaccine-pulsed TCs autonomously generated DCs that induced single-antigen recall responses from autologous naïve and memory CD4+ T lymphocytes, matching study participant immune-status, including BCG responses paralleling donor PPD status, BCG-induced adenosine deaminase (ADA) activity paralleling infant cohorts *in vivo*, and multi-dose HBV antigen-specific responses as demonstrated by lymphoproliferation and TCR sequencing. Overall, our microphysiologic culture method reproduced age- and antigen-specific recall responses to BCG and HBV immunization, closely resembling those observed after a birth immunization of human cohorts *in vivo*, offering for the first time a new approach to early pre-clinical selection of effective age-targeted vaccine candidates.

## Introduction

Infections are most frequent at the extremes of life, especially among newborns, reflecting age-specific differences in immunity (1). Other than provision of clean drinking water, immunization is the most effective means of preventing infections; however, due to distinct early life immunity, many vaccines are also not optimally immunogenic, requiring multiple booster doses to achieve protection. Indeed, despite current vaccination efforts, >2 million neonates (<4 weeks of age) and young infants (<6 months of age) die of preventable infections yearly worldwide. Currently, only Hepatitis B Vaccine (HBV), Oral Poliovirus Vaccine (OPV), and the tuberculosis vaccine Bacille Calmette-Guérin (BCG) have been licensed for neonatal use worldwide (2). Contrasting with the urgency of effective early-life immunization, vaccine development is painfully slow and costly, averaging 15 years to approval (3). Furthermore, early-stages of vaccine development do not typically consider human immune ontogeny and rely heavily on genetically- and immunologically- divergent animal models (4, 5) to select vaccine candidates targeting human infants; a pre-clinical process anticipated to affect end-point effectiveness and therefore, its successful transition to market. Thus, new pre-clinical human immune models informing age-targeted vaccine effectiveness *in vitro* could help accelerate, enhance and de-risk vaccine development, including effective single-dose neonatal vaccines, increasing coverage, reducing infant vulnerability, and yielding major public health benefits (2).

*In vivo*, effectiveness of artificial immunization relies on the necessary detection and tolerance to self-antigens. *In vitro* immune models using exclusively human materials are desirable to enhance the likelihood of achieving single-antigen specific responses to xenogenic antigens provided by vaccines. Efficient protective immunity induced by vaccines requires a microenvironment where a timely interplay of cellular and molecular events enable the capture and processing of rare exogenous antigens by antigen-presenting cells and their subsequent presentation to rare matching naïve lymphocytes at neighboring lymphoid tissues (6). Dendritic Cells (DCs) are the most effective antigen-presenting cells for initiation of T cell immunity (7) and T cells help is considered essential to achieve effective antibody responses (5) to antigenic proteins present in most vaccines. Currently, the use of DCs in human *in vitro* immune models largely rely on cumbersome extractions from tissues or blood, artificial differentiation of monocytes or circulating stem cell precursors using exogenous cytokines (e.g., Granulocyte-Macrophage Colony-Stimulating Factor (GM-CSF) and Interleukin (IL)-4), or the use of immortal DC-like cell lines (8). *In vivo*, most resident lymph-homing DCs develop from blood circulating precursors (e.g., monocytes) after extravasating inside the tissues (9). This autonomous DC differentiation process was first observed *in vitro* by a model of constitutive transendothelial translocation of human monocytes (10) and later confirmed *in vivo* (11); here, DCs traversed the endothelium in an abluminal-to-luminal direction (reverse transendothelial migration) resembling the constitutive tissular egress of dermal DCs en route to the draining lymphatics *in vivo* (12, 13). Tissue niches left behind by migratory DCs *in vivo* are continuously replenished by monocytes extravasating from the general circulation through capillaries (14), small veins consisting of a single-cell endothelium and a basement membrane or interstitium (15). Thus, the endothelium of capillary veins is key to the natural development and relative abundance of tissue-resident monocytes, macrophages (MØ) and DCs, which first encounter pathogens and vaccines *in vivo*.

In light of the physiologic events described above, we took a tissue engineering approach to emulate this natural microanatomy, physiology, and antigenic footprint of a human capillary vein. Naturally consisting of a single-cell endothelial layer and an interstitium, therefore deprived of the cell complexity, migrations, and interactions pertaining to more complex tissues *in vivo*, our tri-dimensional human age-specific vascular-interstitial interphase model recreated the autonomous differentiation and migration behavior of DCs, making it suitable for *in vitro* vaccination studies. While similar prior tissue models have demonstrated autonomous generation of human DCs from extravasating monocytes without the use of cytokines (10, 16–19), these models lacked two key features that may be important for accurate age-specific modeling of human vaccine responses: (1) having an entirely human-derived composition of cells, matrix and fluid phase, desirable as the presence of non-human proteins (e.g., xenogenic bovine matrix, endotoxin) in a human *in vitro* immune testing model may interfere with desired uptake and processing of scarce vaccinal antigens needed to study rare autologous single-antigen specific naïve T cell responses; and (2) including age-specific primary leukocytes and autologous plasma to enable ontogenic assessment of vaccine responses, a key parameter of immunity. A significant novelty of our model is its focus on being as physiologic as possible (e.g., minimal cell manipulation, non-heat-treatment of plasma, no exogenous factors) to maximize the likelihood that *in vitro* results will mirror those *in vivo* thereby enhancing translation. Herein we report the development of age-specific human tissue constructs that enabled natural capture of vaccinal antigens by DCs, and accurate autologous single-antigen specific newborn immune responses to HBV and BCG vaccines, with comparable antigen-specific immunogenicity *in vitro* as observed *in vivo*. This microphysiologic human culture platform offers a new approach to early pre-clinical selection of effective vaccine candidates that could help accelerate, de-risk, and enhance age-specific vaccine development.

## Materials and methods

### Reagents

**Adjuvants**: Adjuvants studied included Aluminum Hydroxide (AlOH; at 5 and 50 μg/mL), Aluminum Phosphate (AlPO, at 2.5 and 25 μg/mL) from Statens Serum Institut (SSI Copenhagen, Denmark). Toll-Like Receptor (TLR) agonists (TLRAs) including TLR2/1A Pam_3_CSK_4_ (Pam3, at 1 and 10 μg/mL), TLR4A 3-O-desacyl-4′-monophosphoryl lipid A (MPL, at 1 and 10 ng/mL) and the TLR7/8A imidazoquinoline Resiquimod (R848, at 5 and 50 μM), all purchased from InvivoGen (San Diego, CA). **Vaccines**: Recombinant Recombivax HB® (HBV, at 1:100, 1:10 and 1:2v/v dilutions) and PNEUMOVAX®23 (PVP, at 1:100 and 1:10v/v dilutions) were purchased from Merck (Whitehouse Station, NJ). PREVNAR-13® (Pneumococcal Conjugate Vaccine or PCV, at 1:100, 1:10, and 1:2v/v dilutions) was purchased from Wyeth Pharmaceuticals Inc. (Pfizer subsidiary; Philadelphia, PA). Pentavalent vaccines given to infants enrolled into the randomized trial in Bissau, Guinea-Bissau (20) were Easyfive™ (Panacea Biotec, India), Quinvaxem® (Berna Biotech Korea Corp), or Pentavac® (Serum Institute of India Pvt. Ltd). BCG Danish strain 1331 (1:100, 1:20 and 1:10v/v dilutions) was purchased from SSI (Copenhagen, Denmark). **Peptides**: Ag-specific challenges were performed using pools of 50% overlapping endotoxin-free peptides based on antigens present in licensed vaccines tested *in vitro* (Supplementary Table 1). Peptide's manufacture was chemical synthesis (Synprosis SA, Fuveau, France). For BCG studies, 28 peptides, ~20-monomers long, encompassing the entire sequence of mycobacterium antigen Ag85A (21), were ordered. For HBV studies, 15 peptides, 10–18 monomers long, encompassing the entire sequence of Hepatitis B virus antigen HBsAg (22), were synthesized. Same number of background control peptides was made using scrambled sequences unrelated to mycobacteria or Hepatitis viruses based on the Basic Local Alignment Search Tool (BLAST®) from the US National Library of Medicine of The National Institutes of Health. Each peptide was individually solubilized at high concentration in DMSO and stored at −20°C, per the manufacturer's recommendations. Before testing, individual peptides were combined to prepare peptide pools stock solutions in plasma-free RPMI media. To remove any potential microparticles, each peptide stock was filtered using a low protein binding, non-pyrogenic, sterile 0.2 mm Supor® membrane filter (PALL Life Sciences Corporation; Port Washington, NY). Peptide stocks concentrations were assessed by the Bicinchoninic Acid protein assay kit (Thermo Scientific Pierce; Rockford, IL). **Other reagents**: Defined Fetal Bovine Serum (FBS) was purchased from Hyclone (Logan, UT). Culture media M199 and RPMI-1640, 1X Hank's Balanced Salt Solution (HBSS), 1X Dulbecco's Phosphate Buffered Solution without calcium or magnesium (DPBS), 0.25% Trypsin-EDTA (100X solution), ultrapure 0.5M EDTA, 16% paraformaldehyde (PFA) solution and 100X Penicillin/Streptomycin/ Glutamine (PSG) solution were purchased from Gibco® (Thermo Fisher Scientific Inc.). Pyrogen-free heparin was purchased from Sagent Pharmaceuticals® (Schaumburg, IL). Collagenase enzyme with no tryptic activity was from Roche Diagnostics. Sodium hydroxide (NaOH 10M), dimethyl sulphoxide (100% ACS DMSO, Hybri-Max®), Trypan Blue stain (0.2%), Haematoxylin-Eosin (H&E), and Glucose-6-Phosphate (G-6-P) were from Sigma-Aldrich (St. Louis, MO). Ficoll-Paque PREMIUM™ was from GE Healthcare Life Sciences (Pittsburgh, PA).

### Human materials

**Newborn and adult blood**: Adult blood (50–300 mL) from healthy study participants (26–45 years old) was collected via peripheral venipuncture after written informed consent in accordance with the Declaration of Helsinki and as approved by the Institutional Review Board (IRB) of Boston Children's Hospital (BCH) (protocol number X07-05-0223). HBV and BCG vaccination history, the most recent mycobacteria Purified Protein Derivative (PPD) Delayed-type hypersensitivity (DTH) status and anti-HBsAg-Ab titers, were obtained for most adult donations. Anti-HBsAg-Ab titers from newborn and adult plasmas were measured at the BCH Clinical Core Laboratory using the Abbott Architect i1000 Immunoassay Analyzer instrument (Abbott Diagnostics, Chicago IL) and their assay kit (#34-5498/R4). While Recombivax HB® manufacturer establishes a correlate of protection of ≥10 mIU/mL (23), the BCH recommendation of ≥12.0 mIU/mL was followed. Newborn blood (50–100 mL) was collected by venipuncture of umbilical cords (mean gestational age 38.9 weeks), immediately after elective Cesarean-section delivery (epidural anesthesia) of de-identified mothers with no record of fever, HIV or other acute or chronic infections, following protocols approved by the local IRBs of The Brigham and Women's Hospital (Protocol #2000P000117/BWH) and the Beth Israel Deaconess Medical Center (Protocol #2011P-000118/BIDMC). Blood samples were drawn into syringes containing pyrogen-free heparin (final concentration 20 units/mL) and processed within 2 h of collection to separate plasma and mononuclear cells. **Heparinized plasma samples from an infant cohort receiving early vs. delayed BCG**: Plasma was obtained from infants enrolled in a randomized trial in Bissau, Guinea-Bissau (West Africa; clinicaltrials.gov: NCT00625482) comparing immunological effects on infants receiving BCG within the first week of life (early BCG) vs. delayed-BCG vaccination (Ø) (20). Eligible newborns weighed <2.50 kg, had no major malformations, had parents who provided informed consent, and were ready to leave the maternity ward. BCG vaccine (SSI, Statens Serum Institut, Copenhagen, Denmark) was normally provided at birth (50 μL, intradermal) for neonates weighing >2.50 kg, otherwise it was postponed until infants gained weight, typically at the time of their subsequent scheduled routine vaccination at 6 weeks of age. Frozen plasmas were shipped from Bissau by air courier under temperature control and were used to assess Adenosine Deaminase (ADA)-1 and ADA2 activities in relation to BCG immunization. Samples from infants who were randomized at >7 days of life to early vs. delayed BCG, as well as samples in the delayed BCG group who had received BCG before donating blood, were excluded of ADA study. All infants received oral polio vaccine (OPV) at birth; none had pentavalent DTPw-HepB-Hib vaccine before 4 weeks of age (recommended at 6, 10, and 14 weeks); most samples studied for ADA activity were from study participants with 1 dose of pentavalent vaccine before the 10-week-old blood donation. **Isolation and cryopreservation of autologous plasma and mononuclear cells**: Blood was centrifuged at 1,200 revolutions per minute (rpm) for 10 min at 24°C to separate plasma and hemocytes. Plasma was centrifuged again at 3,000 rpm for 30 min to generate platelet-poor plasma and then frozen at −20°C until further analysis. Plasma used on studies was never heat-treated. Hemocytes were resuspended in 1x DPBS with 2%v/v heparin to regain the original blood volume. Mononuclear Cells (MCs) were isolated by Ficoll® density gradient centrifugation according to manufacturer's specifications. Newborn cord blood MCs and adult peripheral blood MCs were counted (Trypan Blue exclusion) and cryopreserved at a cell density of 100 million/mL using 1 mL of plasma-free cryopreservation media consisting of 1x DPBS containing 10% DMSO, 44 mg/mL HSA and 2 mM EDTA. MCs were placed inside a pre-cooled (4°C) Mr.Frosty™ freezing container (Nalgene ®, Sigma-Aldrich; St. Louis, MO) according to manufacturer's instructions and rapidly moved to an −80°C freezer. After over-night storage, cell amps were transferred to a liquid nitrogen tank for long-term storage. **Extracellular matrix proteins, albumin and endothelial cells**: Human type I collagen solution (VitroCol®, 3 mg/mL) was purchased from Advanced Biomatrix™ (San Diego, CA). Human Fibronectin was purchased from Biomedical Technologies Inc. (Stoughton, MA). Clinical grade Human Serum Albumin (HSA) was purchased from Octapharma (Lachen, Switzerland). A single-donor HUVEC lot, evaluated for appropriate growth characteristics, was selected for purchase from Lonza™ (Walkersville, MD USA) and used across all of our experiments.

### Endotoxin testing

All human materials and reagents were confirmed to be endotoxin-free (≤0.2 Endotoxin Units/mL at working concentration) with a Kinetic Turbidimetric (KTA) LAL test using the KTA2 reagent and following manufacturer's instructions (Charles River Laboratories International, Inc.; Wilmington, MA). Additionally, some reagents were tested further to ascertain they were biologically inert using a standardized whole blood TNF-α release assay at working concentrations as we have previously described (24).

### Generation of tissue constructs

**Figure 1:**
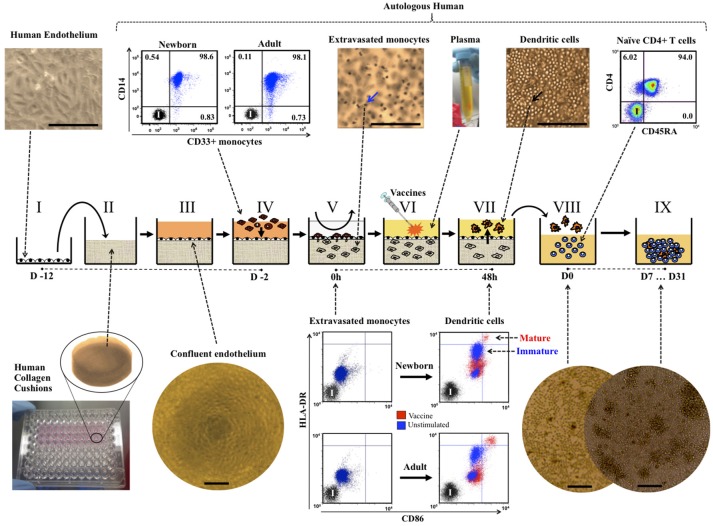
The Tissue Construct (TC) model. Single donor human endothelial cells (I, representative image, 20X) were grown onto cast cushions of human collagen (II) and cultured with human plasma media until reaching quiescent confluency (III, representative image, 4X). Age-specific TCs were created by allowing CD33+ selected newborn or adult monocytes (IV, representative dot plots) to colonize the quiescent confluent TCs. After removal of non-extravasated monocytes (V), age-specific TCs were cultured for 48 h in autologous non-heated plasma +/− adjuvants or vaccines to allow for autonomous dendritic cell (DC) development, maturation and reverse transendothelial migration (V-VII). Age-specific DCs, autonomously developed from TCs stimulated with pediatric vaccines, were co-cultured with autologous untouched naïve CD4+ CD45RA+ T cells (VIII, representative dot plot) and autologous non-heated plasma for seven to 31 days (IX, D7 … D31), as described in methods. Mature (red) and immature (blue) DCs from TCs as defined by the co-expressions levels of HLA-DR and CD86 markers (representative dot plots of initially extravasated monocytes extracted from TCs at 0 h and autologous DCs harvested 48 h later after stimulation). Representative images of co-cultures at Day 0 (D0) and D7 (10X, scale bars = 200 μm). Blue arrow indicates monocytes inside TCs (H&E staining, representative image, 20X). Black arrow indicates DCs accumulated on top of endothelium (phase contrast, representative image, 20X). Scale bars = ~200 μm. I, Isotype controls.

Building from prior tissue engineering efforts (10), our tridimensional TC model consists of a confluent quiescent monolayer of single-donor human umbilical vein endothelial cells (HUVECs) grown over a basement membrane made of human extracellular matrix proteins using human plasma (Figure 1 I–III). This *in vitro* tissue arrangement closely mirrors both the components and architecture of capillary veins *in vivo* (15). **Timing**: The process of creating TCs spanned 10 days before the start of a TC assay (e.g., D-12 to D-2), at which point newborn or adult monocytes were allowed to colonize TCs and adjuvant or vaccine stimulations were begun (D-2). Details are described below along this timeline: **D-12**: Single-donor HUVECs were cultured at 5% CO_2_/37°C in M199 media containing 50% FBS and 1% PSG. HUVECs started culture in 75 cm vented cap tissue culture flasks (Corning Life Sciences; Tewksbury, MA) pre-coated with a 0.5 mg/mL solution of human fibronectin. Excess fibronectin was removed before the addition of the HUVECs. **D-9**: Endotoxin-free human type I collagen cushions were cast in 96-well microtiter plates (Costar round bottom, Thermo Fisher Scientific Inc.), as described earlier (16). Human type I collagen cushion solution was prepared by mixing 10x M199 media, 0.1N NaOH and the human collagen (3 mg/mL) at a proportion 1:5:8, respectively. Seventy microliters of this solution was applied to each of the inner 60 wells of a 96 well flat bottom Falcon® microtiter plate (Becton Dickinson; Bedford, MA) using a repeating dispenser, and the plate incubated at 5% CO_2_/37°C for 24 h. After congealing, cushions were aged by applying a neutralized (with 10N NaOH; pH between 6.5 and 7) and filtered (Acrodisc® unit, at a rate defined by the manufacturer) solution of G-6-P (225 mM in HBSS). Collagen cushions received 50 μL of G-6-P solution using a repeating dispenser and then incubated at 5% CO_2_/37°C for an additional five days. Meanwhile, using Trypsin-EDTA and the same M199 media containing 50% FBS and 1% PSG, the 85–90% confluent HUVEC cultures (as assessed with an inverted microscope) were passed to larger (150 cm) vented cap tissue culture flasks pre-coated with human fibronectin (0.5 mg/mL) and incubated at 5% CO_2_/37°C. **D-4**: Four days before the assay, G-6-P solution was removed out of every cushion by aspiration, 200 μL of HBSS were added to each well of the 96-well plate (including the 36 empty wells around the 60 inner wells with cushions, as an evaporation barrier) prior to returning the plate to the 5% CO_2_/37°C incubator. **D-3**: HUVEC cultures were enzymatically harvested with Trypsin-EDTA, washed twice with 1x DPBS to remove any potential trace of residual FBS and resuspended in fresh M199 media containing 1% PSG and 30% human newborn pooled plasma (previously prepared using small volume leftover plasmas from several study participants). Cells from one confluent T150 flask were resuspended in ~13 mL of media to be dispensed as 100 μL per well (120 wells). Ten minutes before seeding endothelial cells, collagen cushions were prepared by aspirating out the HBSS and adding 20 μL of 0.5 mg/mL human fibronectin. Typically, one T150 flask of confluent HUVECs contained sufficient cells to coat two cushion plates (180 wells). **Assessment of TCS at D-2 before starting an assay**: Monolayer integrity, confluence, and cobblestone morphology of endothelial cells was assessed by inverted microscopy using phase contrast at 4x magnification (Nikon TS100 inverted microscope, Nikon Instruments Inc.; Melville, NY). Only 100%-confluent TCs were used for testing.

### Generation of age-specific human tissue constructs

Age-specificity of TCs was achieved by allowing monocytes from either a newborn or an adult participant to autonomously extravasate inside the TCs and removing non-migrated monocytes after 1.5 h of incubation. Every assay compared responses between one newborn and one adult. Monocytes were positively selected from freshly thawed MCs using magnetic micro-beads covalently linked to anti-CD33 mAbs (MACS® positive selection, Miltenyi; Cambridge, MA). CD33 rather than CD14 was chosen in order to: (a) avoid interference with important antigen-capture molecules (25); (b), avoid induction of non-physiological activation (26); and (c), provide more natural monocyte heterogeneity (27). ~10^5^ monocytes were applied to each TC well and allowed to autonomously extravasate under static conditions for 1.5 h in serum-free M199 media containing 1% PSG and 0.1% HSA at 37°C/5%CO_2_. After this, non-migrated monocytes were removed by gentle aspiration and several consecutive washes with warm 1x DPBS and monocyte-colonized TCs were ready for testing (Figure 1 IV–V). Of note, non-extravasated monocytes do not become DCs after being on the endothelium for 1.5 h, and monocytes that do not reverse transmigrate after 48 h of culture, remain inside TCs as macrophages independently of stimulation (10). To assess monocyte extravasation, TCs were fixed with fresh 10% PFA, stained with H&E and examined with an inverted microscope.

### Assessment of autonomous DC development from age-specific tissue constructs

To enable autonomous generation of age-specific DCs, monocyte-colonized newborn TCs (NTCs) and adult TCs (ATCs) were cultured for 48 h with innate immune stimuli in autologous plasma (Figure 1 VI), a natural source of age-specific immunomodulatory factors (2, 28). After this time, about half of the initially extravasated monocytes become either immature or mature DCs, depending on the type and degree of adjuvant or vaccine stimulation added at step VI, transmigrating back out of the extracellular matrix and across the endothelial layer, in a step that mirrors movement of tissue antigen-presenting cells from tissue sites to the lymphatic system (12, 13). As previously described (10), the only location where TC culture conditions demonstrate high percentage of mature DCs is amongst stimulated reverse transmigrated cells (VII). When TCs are left unstimulated (e.g., vehicle control), reverse transmigrated cells accumulate on the luminal side of the endothelial monolayer at same degree than stimulated TCs but demonstrating very low relative DC maturation. Thus, as with capillary veins *in vivo*, the endothelium of our model is key to the natural development and relative abundance of tissue-resident macrophages and migratory DCs. For practical purposes, since reverse transmigrated cells are a mix of immature and mature DCs, all harvested reverse transmigrated cells will be referred generally as DCs (e.g., DC:T cell co-cultures). **Timing**: The process of stimulating TCs spanned 2 days. Details are described below along this timeline: **D-2**: age-specific (monocyte-colonized) TCs were cultured for 48 h at 37°C/5% CO_2_ in the presence of 100 μL of autologous non-heated plasma, with no additional exogenous cytokines, with or without adjuvants, licensed vaccines or desired controls. Some experiments included conditions in which autologous plasma from the newborn and the adult were swapped to assess the relevance of using autologous plasma, as compared to homologous (adult-newborn swap) or xenogenic (Fetal Bovine Serum). Unstimulated control (Ø) TCs received a volume of sterile aqueous carrier solution equal to corresponding stimulator tested or, when several doses tested, corresponding to the highest concentration tested for that stimulator. In this model, during the 48 h stimulation, monocytes autonomously differentiate into migratory DCs that reverse transmigrate out of TCs, crossing the endothelium in abluminal-to-luminal direction (Reverse Transendothelial migration) to accumulate on the luminal side of the endothelium. **D0**: typically, DCs from 5 to 20 TC replicas/condition were collected by gentle pipetting in warm 1x DPBS and counted with vital staining Trypan Blue following manufacturer's recommendations. **Phenotypic analysis of DCs by flow cytometry**: Autonomous development and maturation of DCs from unstimulated and stimulated age-specific TCs (Figure 1 VII and Supplementary Videos 1–3) was investigated using polychromatic flow cytometry. This technique was also used to assess purity of cell selections. Immunophenotyping used direct-conjugated monoclonal antibodies (mAbs) against CD33-PE, CD14-APC, CD86-PE, HLA-DR-FITC, and CD197-BV421 (Becton Dickinson; Franklin Lakes, New Jersey). Cells were stained for 30 min with mAbs as recommended by manufacturer, rinsed with DPBS and fixed with freshly made 4% PFA solution. Corresponding direct conjugated isotype mAbs and unstained controls were used to determine non-specific binding. Flow cytometry employed a BD LSR-Fortessa™ (Becton Dickinson) and data analyzed using FlowJo® software (Tree Star, Inc.; Ashland, OR) using a single viable gate. To compare the phenotype of cells inside TCs at D-2 with corresponding cells at D0 for a given study participant, cells were retrieved from inside TCs using tryptic-free collagenase digestion, according to manufacturer's recommendations. **Video-microscopy of cells inside TCs**: A trans-perpendicular video of the Tissue Construct after 48 h of culture was generated using series of pictures taken approximately every 20 μm from the top of TCs, moving down (Supplementary Video 1). For this, TCs were fixed with fresh 10% PFA after removal of reverse transmigrated DCs from the luminal side of the endothelium, stained with H&E and then examined with an inverted microscope. A time-lapse video of live monocytes at ~50 μm inside an un-fixed Tissue Construct after 18 h of culture, was generated with pictures taken with the microscope every 30 s for a time span of about 15 min and compressed in ~8 s (Supplementary Video 2). A time-lapse video of live Dendritic Cells projecting dendrites (sampling) at ~50 μm inside an un-fixed Tissue Construct after 24 h of culture, was made with pictures taken every ~15 s for a time span of about 10 min and compressed in ~15 s (Supplementary Video 3). **Analysis of DCs using confocal microscopy**: Confocal microscopy was employed to assess changes in morphology and surface translocation of HLA-DR on DCs stimulated with HBV (1:2v/v) or BCG (1:20v/v). DCs were harvested and transferred to a 24-well flat-bottom tissue-culture plate (Falcon, Becton Dickinson) with BD BioCoat™ round Poly-L-Lysine Coated Glass Coverslips (two 12-mm diameter coverslips per condition) with autologous plasma to continue incubation at 37°C/5% CO_2_ for 1 h and regain natural cell morphology. Coverslips were washed once with warm 1X DPBS and then fixed and permeabilized with BD Citofix/Cytopermt™ (Becton Dickinson). After another wash with BD Washing Buffer, cells were stained with HLA-DR-FITC, DNA dye DraQ5t™ (eBiosciences, Inc.; San Diego, CA) and Alexa Fluor® 594 phalloidin F-actin (Molecular Probes, Inc.; Eugene, OR) in 1x DPBS with 0.5% HSA for 30 min/37°C/dark. Corresponding isotype mAbs served as controls for non-specific background fluorescence. Coverslips were mounted with 50% glycerol in DPBS and sealed with transparent non-florescent nail polish (Thermo Fisher Scientific Inc.). Images were acquired using Slidebook ® software on an Axiovert® 200 m fluorescent microscope (Zeiss; Thornwood, NY), with a 63X Immersion Objective. Slidebook® features allowed for the reconstruction of three-dimensional rotational-ax confocal video-microscopy using acquired stacked pictures (Supplementary Video 4). Also, plane-by-plane confocal video-microscopy reconstructions were made using acquired confocal images taken at focal planes 0.2 μm apart (Supplementary Videos 5, 6). To assess uptake of BCG (*M. bovis*), mycobacteria were labeled with DraQ5™ dye for DNA and lipophilic dye 1,1'-Dioctadecyl-3,3,3',3'-Tetramethylindocarbocyanine Perchlorate (DiI or DiIC18) for bacterial membrane lipids, as previously described (29), prior to addition to TCs. 0.25 μm thick optic focal plane cuts were imaged to verify intracellular presence of bacteria. **ADA activity in plasma after immune stimulation of age-specific TCs**: ADA1 activity was measured in age-specific supernatants (**D0**) from BCG *in vitro* immunized TCs. This technique was also used to determine ADA1 and ADA2 activities in untreated plasma samples from the newborns and adults recruited for our *in vitro* studies, directly after separation from blood, and from plasma samples obtained by a Guinea-Bissau clinical trial comparing birth-BCG vs. delayed-BCG scheduled. ADA1 and ADA2 activities in plasma (Units/Liter) were determined with a chromogenic assay kit (Diazyme Laboratories, Inc.; Poway, CA), in 384-well plates, using recommended controls and calibrator sets and maintaining reagent ratios, as per manufacturer's recommendations. Assay was run in duplicate with or without ADA1 inhibitor erythro-9-(2-Hydroxy-3-nonyl)-adenine hydrochloride (EHNA, 20 μM). Since ADA2 is not EHNA sensitive, ADA2 activity is determined by EHNA-containing wells. Results were read on a Tecan Infinite 200 and ADA1 activity was calculated by subtracting ADA2 activity from total ADA activity, as recommended by the manufacturer. One unit of ADA is defined as the amount of ADA that generates one μmole of inosine from adenosine per min at 37°C.

### Stimulation of autologous lymphocytes by DCs from *in vitro* vaccinated tissue constructs

The stimulatory capacity of autonomous TC-derived DCs toward autologous untouched CD4+ T cells (Figure 1 VIII–IX) was investigated by measuring proliferation and cytokine production after several days of co-culture in the presence of 10% autologous plasma, with no media changes or the addition of exogenous factors. **Timing**: Age-specific TCs were prepared and pulsed (***D-2***) with licensed pediatric vaccines (e.g., HBV, PCV, PVP, or BCG), as previously described. Two days later (**D0**) DCs were harvested and co-cultured for up to seven days with either autologous naïve CD4+ CD45RA+ T cells to assess their capacity to initiate primary responses or with autologous total CD4+ T cells from recruited study participants with positive BCG and/or HBV vaccination history, in order to assess the capacity of our model to detect or re-stimulate memory responses. Untouched CD4+ CD45RA+ naïve T cells and CD4+ pan T cells were isolated by negative selection from freshly thawed MCs using magnetic microbeads (MACS® isolation kit, Miltenyi Biotech GmbH; Bergisch Gladbach, FRG), following the manufacturer's instructions. Lymphocytes were routinely >98% pure, as assessed by multicolor flow cytometry with mAbs CD4-PE, CD3-APC, and CD45RA-FITC (Becton Dickinson; Franklin Lakes, New Jersey). DC:T cell co-cultures were setup in 96-well microtiter plates at ratio 1:10 (5 × 10^3^ DCs + 5 × 10^4^ T cells) in 200 μL of RPMI1640 Media containing 10% autologous plasma and cultured at 37°C/5% CO_2_ with no media changes. **Analysis of autologous lymphoproliferation induced by DCs from** ***in vitro* vaccinated TCs**: Lymphoproliferation was assessed by the H^3^-thymidine incorporation method at various time-points (D3, D5 and D7). Eight hours before analysis, a sample of media was collected and stored at −20°C for cytokine assessment and then, 1 μCi/well of H^3^-Thymidine (PerkinElmer; Waltham, MA) was added to each culture well, including some empty background controls. The whole 96-well-plate with co-cultures were harvested into glass fiber filter mats (PerkinElmer) using a Harvester96 (TomTec; Hamden, CT) and read with a 1450 Microbeta Counter (PerkinElmer). Depending on cell availability, 3 to 14 DC:T cell culture replicas were setup per condition, leaving rows of empty wells in between conditions. Other background controls tested included: unstimulated cultures, naïve T cells that were co-cultured with DCs, naïve T cells co-cultured with live attenuated BCG but no DCs and BCG vaccine alone in regular media with no antibiotics. Counts Per Minute (CPM) were calculated as the mean of all replica co-cultures per harvested condition for each donor. **Cytokines released after immune stimulation of autologous lymphocytes by DCs from** ***in vitro* vaccinated TCs**: Supernatants from DC:T cell co-cultures at D3, D5, and D7 were harvested and stored at −20°C until use. Cytokines were measured after a single freeze-thaw cycle by a fluorometric bead-based array method, using human 17-, 26-, or 29-plex Cytokine/Chemokine panel kits (Millipore; Billerica, MA) and a Luminex Multiplex Instrument (Millipore), following manufacturer's recommendations. This technique was also used to measure cytokines released in cultures after an Ag85A-specific peptide challenge. Panel of analytes included IL-1ra, IL-1α, IL-1β, IL-2, IL-3, IL-4, IL-5, IL-6, IL-7, IL-8 (CXCL8), IL-10, IL-12p40, IL-12p70, IL-13, IL-15, IL-17, TNF-α, TNF-β, IFN-α2, IFN-γ, IP-10, CCL2, G-CSF, GM-CSF, MIP-1α, MIP-1β, EGF, Eotaxin (CCL11), and VEGF.

### Autologous Ag85A-specific lymphocyte challenge responses induced by BCG *in vitro* immunization

**Timing**: Age-specific TCs were prepared and pulsed with licensed pediatric vaccine BCG or left unstimulated, as previously described. At **D0**, DCs were harvested and co-cultured with autologous naïve CD4+ CD45RA+ naïve T cells for up to 15 days. As before, all DC:T cell co-cultures were setup in 96-well microtiter plates at ratio 1:10 (5 × 10^3^ DCs + 5 × 10^4^ T cells) in 200 μL of RPMI1640 Media containing 10% autologous plasma, with no media changes. **D15**: Resting T cells were harvested, washed, counted and re-plated (5 × 10^3^ cells/well) in 10% autologous plasma media to undergo further culture-challenge with 5 × 10^4^ freshly selected autologous CD33+ monocytes (10:1 monocyte:T cell ratio) and a pool of peptides encompassing the entire sequence of mycobacteria antigen Ag85A. Peptides and monocytes were not pre-incubated and no media changes or additives were provided during culture. **D20, D22 and D25**: At days 20, 22, and 25, cells and supernatants were analyzed for proliferation and cytokines, respectively. **Autologous Ag85A-challenge lymphoproliferation induced by** ***in vitro* immunization with BCG**: Split T cells (**D15**) were either challenged with a pool of 28 Ag85A peptides (Ag-specific, 560 μg/mL) or with a pool of 28 corresponding scrambled sequences (background control, 560 μg/mL). Single-peptide final test concentration was ~20 μg/mL. Other specificity controls included were split T cells with no challenge, split T cells challenged with peptides but no monocytes and Ag-specific challenged autologous naïve T cells never exposed to DCs from BCG-stimulated TCs. Lymphoproliferation in response to challenges was assessed by the H^3^-thymidine incorporation method at D20, D22, and D25, with as many replica wells per condition as possible, as previously described. **Cytokines induced by Ag85A-challenge after BCG** ***in vitro* immunization**: Supernatants from D25 were harvested and stored at −20°C until use. Cytokines were analyzed by multiplex cytokine bead array, as previously described.

### Autologous HBsAg-specific lymphocyte challenge responses induced by HBV *in vitro* immunization

**Timing**: Age-specific TCs were prepared and pulsed (**D-2**) with licensed pediatric vaccine HBV (1:100v/v dilution), as previously described. At **D0**, DCs were harvested and co-cultured at 37°C/5% CO_2_ with autologous total CD4+ T cells in 96-well microtiter plates at ratio 1:10 (5 × 10^3^ DCs + 5 × 10^4^ T cells) in 200 μL of RPMI-1640 Media containing 10% autologous plasma. To model the second immunization of HBV, as given *in vivo*, a second set of age-specific TCs was started at **D5**, using autologous monocytes from same two participants used at **D-2**. These new TCs were vaccinated *in vitro* with same lot and dose of HBV (1:100v/v), but now using two kinds of plasmas: (a) autologous plasma; and (b), half autologous plasma mixed with half plasma from the opposite age-group participant being tested on that same assay (1:1v/v mix of plasmas). At **D7**, half of media (100 μL) of the ongoing DC:T cell co-culture was removed by gentle aspiration to provide room for the second round of autologous HBV-stimulated DCs (Boost). Boosting DCs were harvested from the new set of TCs and supplied to ongoing co-cultures in 100 μL of corresponding 10% autologous plasma media or 10% plasma from the opposite age-group participant being tested on that same assay; such that, when combined with remaining autologous co-culture media produced a final 10% of 1:1v/v mixed plasmas. Thus, from **D7** onward for each age-group participant on that assay half the boost DC:T cell co-cultures will have autologous plasma and the other half will have mixed plasma. At **D21**, resting T cells were harvested, washed, counted, and re-plated (5 × 10^3^ cells/well) in corresponding plasma media (same as used at **D7**) to undergo further culture (CHALLENGE) with 5 × 10^4^ freshly selected autologous CD33+ monocytes (10:1 monocyte:T cell ratio) and a pool of peptides encompassing the entire sequence of HBV antigen HBsAg. Peptides and monocytes were not pre-incubated. At **D31** selected samples were analyzed for proliferation and T cell Receptor (TCR) sequencing. **Autologous HBsAg-challenge lymphoproliferation by HBV** ***in vitro* immunization**: Split T cells (**D21**) from each age-group participant were either challenged with a pool of 15 HBsAg peptides (300 μg/mL) or with a pool of 15 corresponding scrambled sequences (background control, 300 μg/mL). Single-peptide final test concentration was ~20 μg/mL. Lymphoproliferation in response to challenges was assessed by the H^3^-thymidine incorporation method at **D31**, with as many replica wells per condition as possible, as previously described. All adult blood used for these experiments was from participants with positive HBV vaccination history. Plasma HBsAg-Ab titers were investigated after conclusion of the study (Supplementary Table 2). Inter-assay peptide-challenge proliferation reproducibility was retrospectively investigated after realizing the unplanned re-usage of blood from 3 adult participants; these donations occurred with >1 year span in between them (Supplementary Figure 1). **HBsAg-challenge expansion of T cell clones associated to HBV** ***in vitro* immunization**: Cells from three HBsAg-challenge experiments, each containing one newborn tested side-by-side to one adult, were taken from seven time-points/conditions detailed at design schematics (**Figure 6A**, indicated as ①, ②, ③, ④, ⑤, ⑥, and ⑦). Fast-frozen cell pellets were shipped to Adaptive Biotechnologies® Inc. (Seattle, WA) where genomic DNA was prepared using the Qiagen® DNeasy Blood & Tissue Kit (Germantown, MD), according to the manufacturer's instructions. Samples were quantified using Dropsense96 (TRINEAN NV; Gentbrugge, Belgium), diluted in buffer to a standard concentration for library preparation prior to high-throughput sequencing of the TCRβ complementarity-determining region 3 (CDR3). Sequences were amplified from genomic DNA using a two-step, bias-controlled multiplex PCR approach (30). First PCR amplified the hypervariable CDR3 of the immune receptor locus using forward and reverse amplification primers specific for every V and J gene segments. Then, a second PCR added “proprietary barcode” and “Illumina adapter” sequences (Illumina®, Inc.; San Diego, CA). CDR3 libraries were sequenced on an Illumina instrument according to the manufacturer's instructions. Raw sequence reads were demultiplexed according to Adaptive's proprietary barcode sequences. Demultiplexed reads were then further processed to: (a) remove adapter and primer sequences; (b), identify and correct for technical errors introduced through PCR and sequencing; and (c), remove primer dimer, germline and other contaminant sequences. Data was filtered and clustered using the relative frequency ratio between similar clones and a modified nearest-neighbor algorithm, to merge closely related sequences. The resulting sequences were sufficient to enable annotation of the V(N)D(N)J genes, constituting each unique CDR3, and the predicted translation of the encoded CDR3 amino acid sequences. V, D and J gene definitions were based on annotation in accordance with the ImMunoGeneTics (IMGT) database. All rearrangements having an in-frame V and J gene within the CDR3 region (e.g., relative to the conserved cysteine and phenylalanine, respectively), and containing no stop codons, were considered productive, i.e., capable of being part of a functional antigen-recognition pocket region of a TCR. The set of observed biological 87 nucleotides long TCRβ CDR3 sequences were normalized to correct for residual multiplex PCR amplification bias and quantified against a set of synthetic TCRβ CDR3 sequence analogs (30). The number and frequency of productive rearranged nucleotide TCR sequences for each condition and donor, each one likely reflecting a single-donor T cell with a functional TCR, was formatted and exported for further analysis using the ImmunoSEQ® Analyzer (Adaptive Biotechnologies; Seattle, WA). The sum of frequencies of productively rearranged non-shared TCRs from each peptide-challenge sample at **D31** (③, ④, ⑥, and ⑦), that were also detected at corresponding **D21** HBV PRIME-BOOST sample (② or ⑤), were plotted side-by-side to evaluate if viable HBV stimulated (**D0**) T cell clones had been preferentially maintained (survival) or numerically increased by HBsAg-specific challenge (HBV-associated TCRs) after 31 days of culture.

### Statistical analysis

Typically, each assay had a pair of age-specific study participants (one newborn and one adult), tested side-by-side on each condition. *P* ≤ 0.05 were considered statistically significant and typically denoted as ^*^ < 0.05, ^**^ < 0.01, and ^***^ < 0.001. Results reported are the mean of multiple technical replicates. Unless otherwise stated, descriptive statistics are reported as the mean of results produced by each study participant age group ± standard deviation. Paired and Unpaired T-tests were employed to compare matched observations (e.g., conditions and time-points) and different participants (e.g., age groups), respectively. For the Ag85A-specific challenge, counts per minute (CPMs) from D20,–22, and –25 of assay were normalized by subtracting nonspecific proliferation from Ag85A-corresponding scrambled sequence peptides, log-transformed to achieve normality (Kolmogorov-Smirnov test) and then the parametric paired *t*-test, two-tailed, was applied to compare Ag85A-specific vs. scrambled peptides. For Stimulation Index (SI) assays, one-sample paired T-test against the hypothetical value of 1.00 was used to compare normalized data (GraphPad Prism 4 software, GraphPad Software Inc.). For analysis of ADA activity in plasma from Guinea-Bissau randomized trial, a sensitivity analysis using linear regression was additionally performed to control for any potential effect of birth weight (Stata12, StataCorp, TX, USA). TCR analysis was performed on three HBV experiments (non-consanguineous newborns and adults; *N* = 3 per group) each with seven different time points/conditions. As all TCs for HBV experiments used the same primary single-donor HUVECs that could potentially represent a carry-over contaminant to DC:T cell co-cultures, we sequenced a sample of untreated HUVECs to validate and control TCR analysis. TCR sequences, with their relative frequencies, from the 43 samples (42 test samples plus 1 control HUVEC sample) were exported using the ImmunoSEQ® Analyzer tool (Adaptive Biotechnologies; Seattle, WA). No endothelial TCR sequences were detected among the 164,738 productive rearranged TCR sequences generated by the 42 test samples. Test conditions from non-consanguineous participants were pooled per age group (newborns and adults; *N* = 3). To study the relationship between the T cells resulting from the HBV *in vitro* immunization process (D21) and those resulting from the Ag-challenge (D31), productive re-arranged TCR sequences from samples ② and ⑤ (D21) were located at samples ③, ④, ⑥, and ⑦ (D31). Statistical analyses of TCR numbers and frequencies were performed for these four resulting datasets (②-③, ②-④, ⑤-⑥, and ⑤-⑦) for each age group. Only mutually exclusive TCRs, not overlapping between peptide types in a given plasma condition, were analyzed. As datasets were not normally distributed, *P* values were calculated as the minimum value of two non-parametric tests (Mann-Whitney U test and One-sample sign test, one-sided). Paired observations were used when comparing conditions from the same study participant.

## Results

### Autonomous generation of age-specific tissue construct-derived mature DCs in response to vaccines

**Figure 2:**
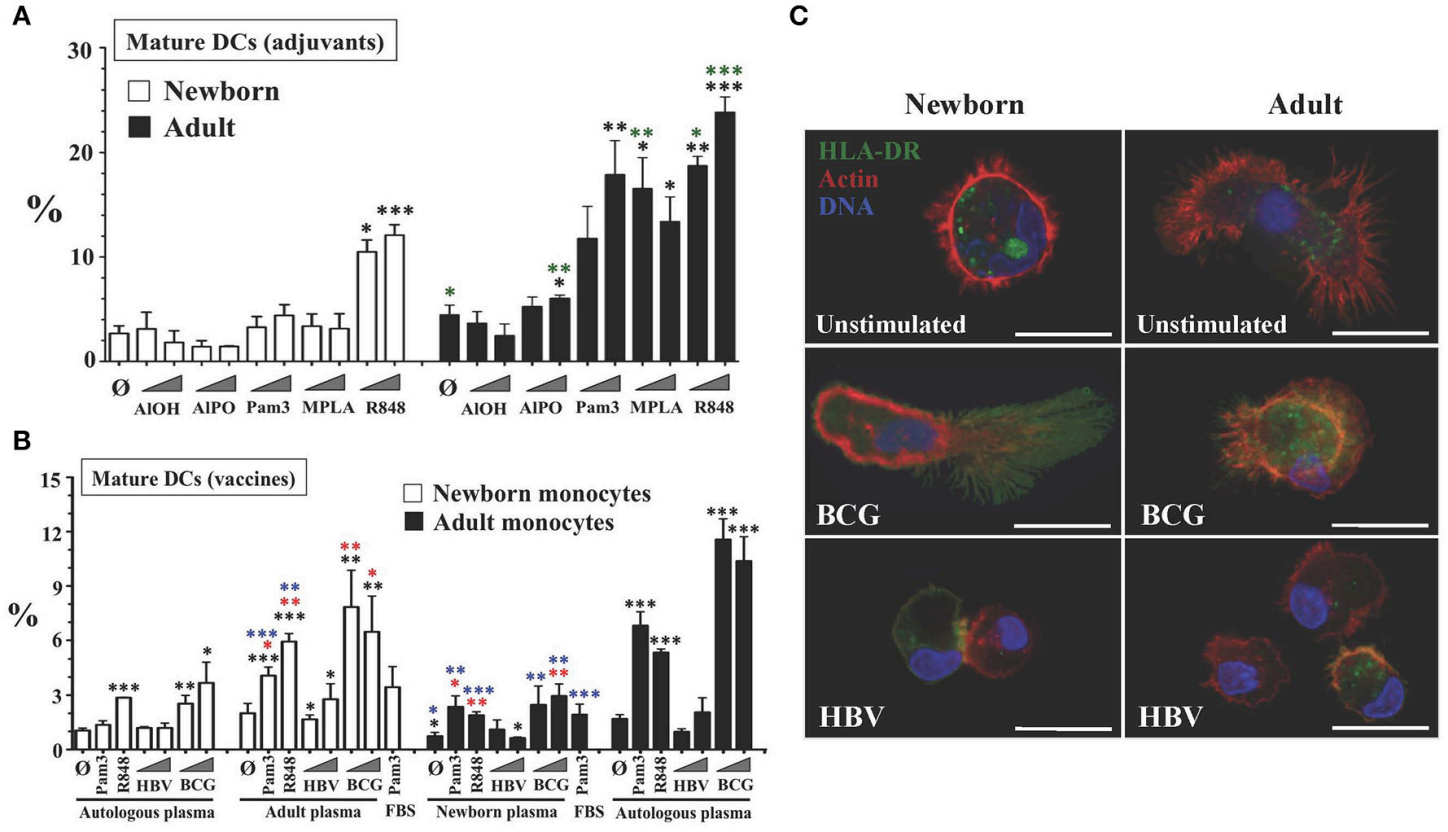
Tissue constructs enable age- and stimulus-specific generation of autonomously generated newborn and adult DCs by adjuvants and licensed vaccines. **(A)** Age-specific maturation of autonomously generated DCs after adjuvant stimulation of TCs. Percentage of viable mature DCs (HLA-DR^hi^/CD86^hi^/CD197^+^/CD14^lo/−^) from age-specific TCs stimulated with vaccinal adjuvants: Aluminum hydroxide (AlOH, at 2.5 and 25 μg/mL), Aluminum Phosphate (AlPO, at 2.5 and 25 μg/mL), Pam_3_CSK_4_ (Pam3, at 1 and 10 μg/mL), 3-O-desacyl-4′-monophosphoryl lipid A (MPLA, at 1ng/mL and 10ng/mL) and Resiquimod (R848, at 5 μM and 50 μM). Triangles indicate low to high concentration. Black stars compared every condition to unstimulated autologous plasma controls, per age group. Green stars compared age groups for same test and condition. *N* = 3-10 donors per age group with ≥7 technical replicas per condition. **(B)** Age-specific maturation of autonomously generated DCs after vaccine and plasma type stimulation of TCs. Percentage of viable mature DCs (HLA-DR^hi^/CD86^hi^/CD197^+^/CD14^lo/−^) from age-specific TCs stimulated by weak newborn stimulus Pam3 (10 μg/mL), robust newborn stimulus R848 (50 μM) and HBV (1:10v/v and 1:2v/v dilutions) and BCG (1:20v/v and 1:10v/v dilutions) vaccines. Triangles indicate low to high concentration. Each experiment included conditions in which autologous plasma from the newborn and the adult were swapped, as described in Methods. FBS was only tested for Pam3. Red stars compared every condition to unstimulated control (Ø), per plasma type group; black stars compared every condition to unstimulated autologous plasma controls, per age group; blue stars compared the effect of plasma type on each condition, per age group. *N* = 3-10 donors per age group with ≥7 technical replicas per condition. **(C)** Surface translocation of cytoplasmic HLA-DR on autonomously generated DCs after HBV or BCG vaccine stimulation of age-specific TCs. Cytoplasmic stored HLA-DR class II molecules are seen in green, F-actin filaments (cytoskeleton) in red and DNA in blue (representative Confocal-microscopy images, scale bar = 10 μm). *P* = * < 0.05; ** < 0.01; *** < 0.001.

Reverse transmigrated leukocyte fractions from NTCs and ATCs stimulated with low-to-high sublethal concentrations of five well-known hydrosoluble adjuvants and two licensed newborn vaccines were analyzed by flow cytometry for the presence of mature DCs. Conventional surface phenotype of mature human DCs (9) includes the co-expression of high levels of antigen-presenting molecule HLA-DR and costimulatory molecule CD86 (Figure 1 VII), *de novo* expression of mature DC marker CD197 (formerly CCR7), and negligible levels of monocyte marker CD14 (HLA-DR^hi^/CD86^hi^/CD197^+^/CD14^lo/−^). Relative percentages of viable mature DC events (Figures 2A,B), as well as surface expression levels (Mean Fluorescent Intensities) for these markers (Supplementary Figure 2), were analyzed for adjuvants such as Alum, either as hydroxide (Al-OH) or as Phosphate (Al-PO), TLRAs including bacterial lipopeptide Pam_3_CSK_4_ (Pam_3_; TLR1/2A), Monophosphoryl Lipid A (MPLA; TLR4A), and resiquimod (R848; TLR7/8A), as well as the licensed vaccines HBV and BCG. Viability of reverse transmigrated cells from stimulated TCs was not significantly different between age groups, with means ranging 73.6–84.83% in NTCs and 83.8–92.6% in ATCs. Reverse transmigrated cells from unstimulated TCs from both age groups showed higher surface expression of CD86 and HLA-DR than initially extravasated autologous monocytes, reflecting on their autonomous differentiation into immature DCs (10). DC maturation is only achieved after efficient immune stimulation. Of note, the number of mature DCs was typically higher in adults than newborns in response to most adjuvants and BCG vaccine. Consistent with the known ontogeny of human neonatal leukocytes in responding weakly to certain adjuvants, and reflecting on inhibitory newborn plasma factors, including adenosine (2, 31), of all stimuli tested only R848 and BCG vaccine induced mature DCs from NTCs. To assess the microphysiological relevance of culturing cells in autologous plasma (Figure 2B and Supplementary Figure 2), we compared DC maturation in response to the relatively weak activator of human neonatal leukocytes TLR2A Pam3 and the robust activator TLR7/8A R848 (32), as well as HBV and BCG vaccines, under different plasma conditions. Consistent with previously reported immunosuppressive effects of neonatal plasma toward TLR-mediated pro-inflammatory signaling in whole blood cultures, especially toward TLR2As (24), the TLR2A Pam3 was less active in the presence of autologous intact newborn plasma, while substitution with adult plasma enhanced adjuvant-induced maturation of newborn DCs, especially in response to Pam3. In contrast, newborn plasma suppressed stimulus-induced maturation of adult DCs. Consistently, Fetal Bovine Serum (FBS) limited the Pam3 response of adult cells similar to the effect of human newborn plasma. Confocal microscopy of reverse transmigrated cells from BCG-stimulated TCs (Figure 2C and Supplementary Videos 4–6) demonstrated a classic DC morphology and maturation as indicated by surface translocation of HLA-DR molecules (green), initially stored in Class-II vesicles of immature DCs (33). Internalization of BCG *M. bovis* by newborn and adult reverse transmigrated cells (Supplementary Figure 3) induced a nearly homogeneous DC maturation (Figure 2C), while in contrast the alum-based HBV generated a polarized mix of mature and immature DCs.

### DCs from vaccine-pulsed tissue constructs induce autologous CD4+ T cell responses

**Figure 3:**
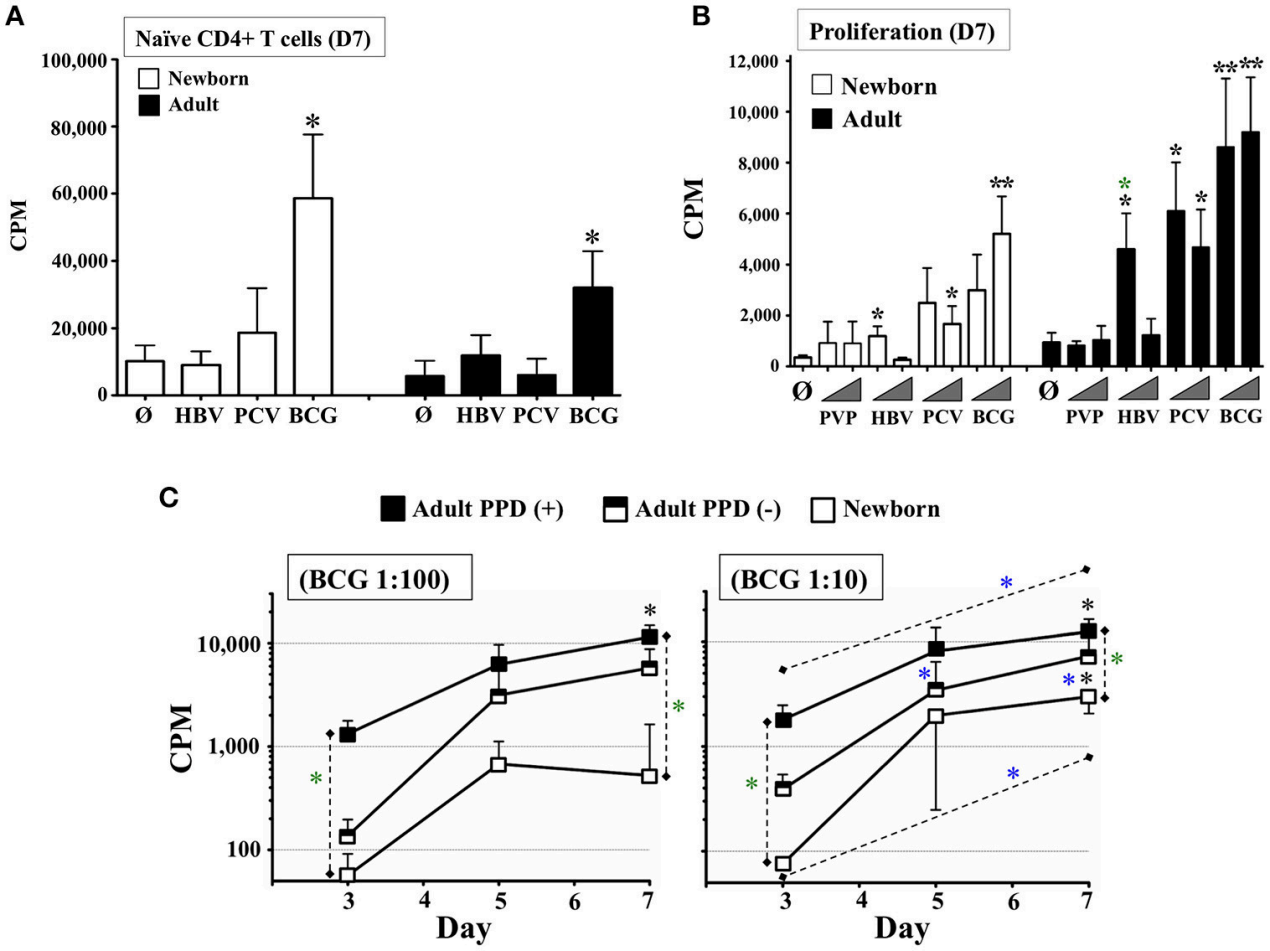
Autonomously derived DCs from tissue constructs pulsed with licensed pediatric vaccines induce autologous age-specific T cell proliferation. **(A)** Autologous naïve T cell proliferation induced by DCs autonomously generated from age-specific TCs stimulated with pediatric vaccines HBV, PCV, and BCG. Proliferation of Naïve CD4+ CD45RA+ T cells (Counts Per Minute or CPM) was assessed at day 7 (D7). BCG was used at 1:20v/v dilution; HBV and PCV were tested at 1:2v/v dilution. *N* = 5–7 participants/age group, per condition. Black stars compared vaccines vs. unstimulated controls (Ø) on same age group. **(B)** Autologous total T cell proliferation induced by DCs autonomously generated from age-specific TCs stimulated with pediatric vaccines PVP, HBV, PCV, and BCG. Lymphoproliferation (CPM) was assessed at day 7 (D7). T cells were total CD4+. PVP, HBV, PCV and BCG vaccines were tested at equal concentrations. Triangles indicate low (1:100v/v) and high (1:10v/v) test concentrations. *N* = 3–9 donors per age group per condition, with ≥7 technical replicas per data point. Recruited adults had positive history of BCG and HBV vaccinations. Black stars compared vaccines vs. unstimulated controls (Ø) on same age group; green stars compared same condition between age groups. **(C)** Cognate lymphoproliferation induced by BCG *in vitro* immunization. Proliferation of CD4+ T cells (CPM) induced by autologous DCs from age-specific TCs stimulated with BCG (1:100v/v and 1:10v/v dilutions) was assessed at days 3, 5, and 7. Recruited adults had positive history of BCG immunization and recent DTH assessment as PPD+ or PPD–. Corresponding unstimulated background controls were individually subtracted to compare dose and time between naïve and immunized participants. *N* = 5 newborns, 4 PPD– adults and 5 PPD+ adults. Black stars compared BCG vs. theoretical mean 0.0; green stars compared same condition between participants; blue stars compared the effect of BCG dose on a given participant/time or BCG time-point for the same participant/dose. *P* = * < 0.05; ** < 0.01.

The ability of BCG vaccine to induce apparently effective mature DCs from NTCs was consistent with its efficacy *in vivo* (2) and enabled us to investigate autologous adaptive responses *in vitro*. Thus, we focused our attention on modeling the type of lymphocyte responses induced by this vaccine *in vivo* where a single neonatal dose of BCG induces T-cell proliferation and cytokines (2). Indeed, CD4+ T cells play an important role in host defense against *Mycobacteria* spp., as evidenced by susceptibility of patients with HIV or primary immunodeficiencies such as SCID or IL-12- or IFN-γ-deficiency (34, 35). Accordingly, we evaluated the functional capacity of age-specific TC-derived DCs (reverse transmigrated cells) to induce proliferation of naïve untouched autologous T helper cells. DCs from BCG-stimulated TCs were harvested and co-cultured with autologous CD3+/CD4+/CD45RA+ T cells (36) to measure proliferation (Figure 3A), as described in Methods. Alum-adjuvanted vaccines HBV and PCV, previously studied in human newborns *in vivo* (37), were also tested in parallel, as reference. Our approach was to co-culture TC-derived DCs and T cells under autologous plasma for 7–15 days without media changes to avoid disrupting naturally formed cytokine microgradients (38). Purified CD33+ monocytes enabled the harvest of pure myeloid DCs and a fixed DC:T cell co-stimulation ratio enabled direct functional comparison between age groups and conditions. To test *in vitro* concentrations matching the relative vaccine antigen/adjuvant contribution of a single newborn dose *in vivo*, as above, HBV and PCV (*in vivo* dose of 500 μL each) were tested initially at dilution 1:2 v/v, while BCG vaccine was tested at dilution 1:20 v/v (*in vivo* dose of 50 μL each). Consistent with DC maturation results, only DCs from BCG-pulsed NTCs and ATCs stimulated significant proliferation of autologous naïve T cells at Day 7 (D7) of culture. BCG results were confirmed with a seven-day time-course proliferation with background controls including unstimulated cultures, naïve T cells alone (no DCs), naïve T cells plus live attenuated BCG (no DCs), and BCG vaccine alone with no antibiotics (Supplementary Figure 4).

We next characterized vaccine-induced production of Th-polarizing cytokines in supernatants from previous co-cultures (Supplementary Table 3). Paralleling lymphoproliferation, adult co-cultures vaccinated *in vitro* with BCG produced significantly higher concentrations of IL-1α, IL-6, IL-7, CXCL8, TNF-β, IFN-γ, and GM-CSF in response to BCG vs. matched unstimulated controls. Newborn BCG co-cultures produced an even wider variety of cytokines, including IL-1β, IL-2, IL-5, IL-6, IL-7, CXCL8, IL-10, IL-12p70, IL-13, IL-15, IL-17, TNF-α, TNF-β, IFN-α2, IFN-γ, GM-CSF, and CCL11. All of these corresponded with cytokines induced by recall challenge of infant leukocytes after neonatal BCG immunization *in vivo*, including IL-1β, IL-2, IL-5, IL-6, IL-10, IL-12p70, IL-13, IL-17, TNF-α, and IFN-γ (20, 34, 35, 39–55) (Supplementary Table 4). In contrast to BCG, HBV and PCV co-cultures produced a markedly smaller array of cytokines and chemokines at relatively lower concentrations. Adult HBV co-cultures showed significant levels of CCL2, while newborn HBV co-cultures produced significant IL-7, CXCL8, IFN-α2, and CCL11 (CCL2 was high but without statistical power). Despite being measured, IL-5, IL-13, or IFN-γ cytokines were not significant *in vitro*, an aspect that is in agreement with HBsAg-recall challenge responses from infants receiving only one birth dose of HBV *in vivo* (Supplementary Table 4) (56). Adult PCV co-cultures generated significant levels of IL-1α, IL-6, CXCL8, IFN-γ, GM-CSF, CCL2, and CCL11; while newborn PCV co-cultures demonstrated production of IL-1β, IL-6, IL-7, CXCL8, IL-10, IL-12p70, IFN-α2, IFN-γ, and CCL11 (IL-13 and CCL2 were high but without statistical power). *In vivo*, recall challenge of blood with PCV-carrier protein Diphtheria CRM197 induced IL-1β, IL-5, IL-6, IL-9, IL-10, IL-12, IL-13, TNF-α, and IFN-γ from infants finishing a three dose PCV series (birth, 1, and 2 months of age) (Supplementary Table 4) (37).

Subsequently, we tested the capacity of TCs to sense donor's immunological memory to vaccines by using negatively selected autologous total CD4+ T cells from adults with recent Delayed-Type Hypersensitivity (DTH) test against the mycobacterial Purified Protein Derivative (PPD or Tuberculin), as well as documented HBV vaccination status and active immunity to HBV according to their Hepatitis B surface Antigen (HBsAg) antibody (Ab) levels (>12.0 mIU/mL). CD4+ lymphocytes were co-cultured for 7 days with autologous DCs from TCs immunized *in vitro* with BCG, HBV, PCV, and the T-cell independent (57) polysaccharide vaccine, Pneumococcal Vaccine Polyvalent (PVP, Pneumovax®23), included for comparison to the T-dependent PCV vaccine response (58). After 7 days of culture (Figure 3B), adult and newborn CD4+ T cells proliferated significantly in response to at least one of the two concentrations tested (1:100v/v and 1:10v/v dilutions) for every vaccine except PVP. Mirroring the requirement for at least three infant doses of HBV (at birth, 2, and 6 months of age) *in vivo* to try to achieve seroprotection (2), neonatal TC HBV responses were detectable vs. unstimulated controls, but were relatively lower than those from HBV-immunized adults. The strong neonatal lymphoproliferative response to BCG, coupled with a lack of a neonatal lymphoproliferation to PVP, mirrored observations by clinical studies, further supporting the hypothesis that the TC reflects immune responses relevant *in vivo* (23, 59). To our knowledge, no studies have assessed neonatal T cell responses after a single dose of PCV vaccine *in vivo*, precluding direct comparison with the NTC readout for this vaccine. However, the significant PCV response from ATCs was in agreement with the expected age-dependent immune-competency of adults against *Streptococcus pneumoniae* (2). BCG results were further validated by measuring lymphoproliferation through time and dose on adult donors grouped as PPD+ and PPD- (Figure 3C). CD4+ lymphocytes stimulated by autologous DCs from TCs immunized *in vitro* with BCG, proliferated in a kinetic- and dose-dependent (1:100v/v vs. 1:10v/v) manner above unstimulated controls, with extent of proliferation mirroring the relative immunological memory of the participants tested.

### Autologous antigen-specific recall responses by age-specific DCs from BCG-immunized tissue constructs

**Figure 4:**
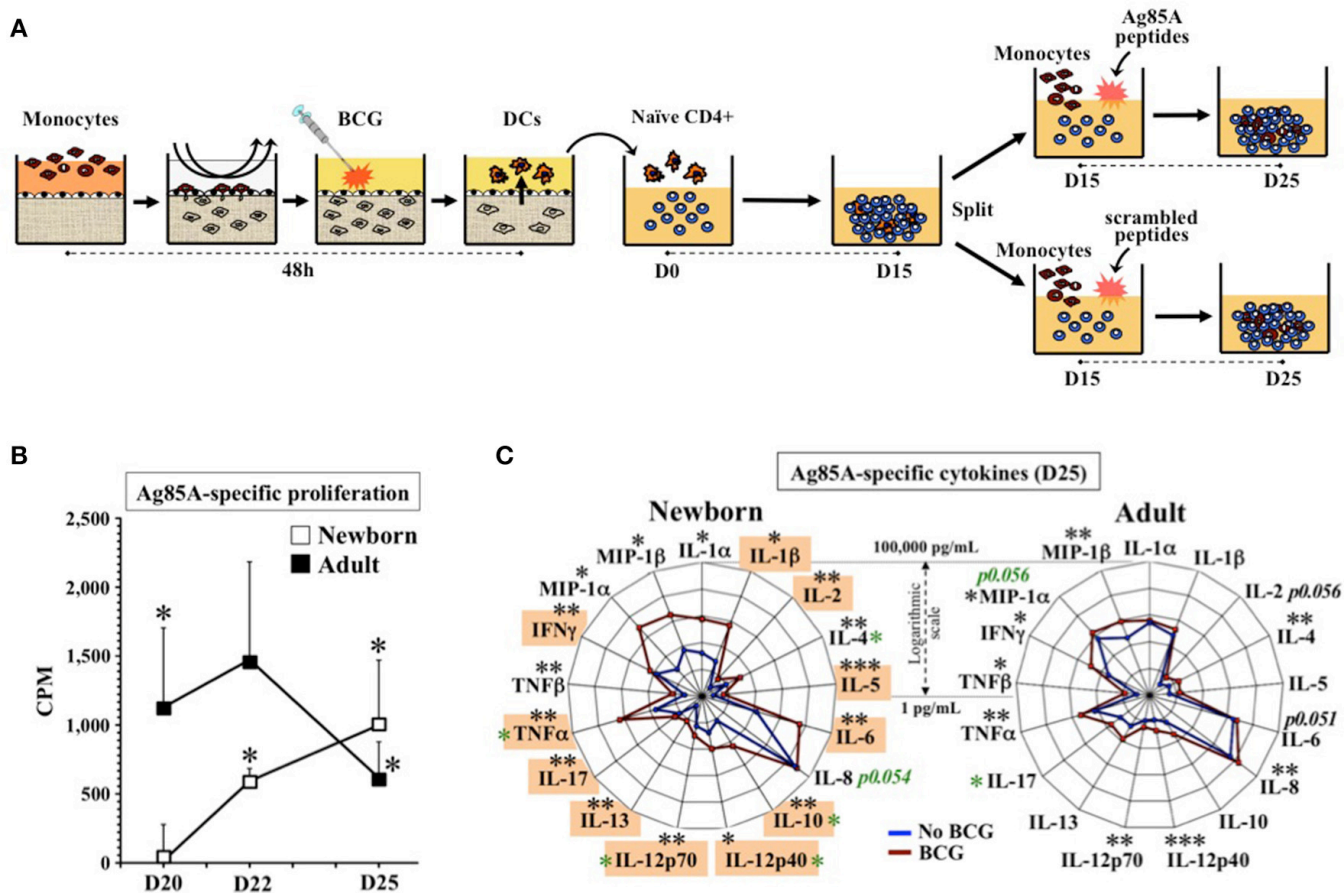
Human tissue constructs model autologous age- and antigen 85A-specific responses after BCG immunization *in vitro*. **(A)** Assay design to assess autologous Ag85A-specific recall-responses after BCG newborn *in vitro* immunization. DCs from age-specific TCs stimulated with BCG (1:10v/v) were co-cultured with autologous naïve CD4+ T cells for 15 days (D15) and split (5,000 cells/well) to undergo antigen challenge for 10 more days (D25) using autologous monocytes with either a peptide pool of antigen-specific Ag85A or a scrambled sequences control. **(B)** Autologous newborn Ag85A-specific recall-proliferation induced by BCG *in vitro* immunization. Ag85A-specific lymphoproliferation (CPM) at days 20, 22, and 25 after peptide-challenge following assay design **(A)**, as described in methods. Corresponding non-specific background from scrambled sequence peptides were individually subtracted to Ag85A peptides CPMs (*N* = 6–7 participants/age group). Black stars compared Ag85A-specific vs. theoretical mean 0.0. **(C)** Autologous newborn Ag85A-specific recall-cytokines induced by BCG *in vitro* immunization. Cytokine profile induced by Ag85A-peptides challenge following assay design **(A)**, as described in methods. DCs from age-specific TCs were either stimulated with BCG (1:10v/v, red line) or left unstimulated (blue line) before co-cultured with autologous naïve CD4+ T cells for 15 days (D15) and split (5,000 cells/well) to undergo antigen challenge for 10 more days (*N* = 6–7 participants per age group). Orange boxes highlight *in vitro* cytokines matching those induced *in vivo* by recall antigens in infants vaccinated at birth with BCG (20, 34, 35, 39–55) (Supplementary Table 4). Black stars compared BCG vs. no BCG; green stars compared conditions between age groups. *P* = * < 0.05; ** < 0.01; *** < 0.001.

Next we assessed whether TC-derived DCs could stimulate single antigen-specific responses in autologous naïve newborn CD4+ T cells after *in vitro* immunization with BCG vaccine. TCs were left unstimulated or immunized with BCG (1:10v/v) in autologous plasma; autonomously generated DCs were then co-cultured with autologous untouched naïve CD4+/CD45RA+ T lymphocytes at a fixed ratio in the presence of autologous plasma for up to 15 days, without media changes. At Day 15, rested T cells were split (to 5,000 cells/well) and challenged for 10 days more with autologous CD33+ monocytes and either scramble control peptides or peptides spanning the entire protein sequence of BCG antigen Ag85A (Figure 4A) (60). At D25, Ag85A-challenge induced significant proliferation of newborn and adult cultures above that of scramble-challenge, unchallenged T cells or Ag85A-challenged autologous naïve T cells that never received BCG-stimulated DCs (Figure 4B). Consistent with their observed Ag85A-specific proliferation, newborn D25 supernatants demonstrated significant levels of IL-1α, IL-1β, IL-2, IL-4, IL-5, IL-6, IL-10, IL-12p40, IL-12p70, IL-13, IL-17, TNF-α, TNF-β, IFN-γ, MIP-1α, and MIP-1β (Figure 4C); and adult D25 supernatants produced IL-4, CXCL8, IL-12p40, IL-12p70, TNF-α, TNF-β, IFN-γ, MIP-1α, and MIP-1β, as compared to the non-vaccinated controls. Newborn cultures produced greater concentrations of IL-4, IL-12p40, IL-12p70, and TNF-α, than adults, while adult co-cultures produced greater concentrations of IL-17. Eleven out of the 17 effector cytokines found on newborn DC:T cell co-cultures vaccinated *in vitro* with BCG (Supplementary Table 3) were also found after Ag85A-specific challenge, including IL-1β, IL-2, IL-5, IL-6, IL-10, IL-12p70, IL-13, IL-17, TNF-α, TNF-β, and IFN-γ. Of note, of these 11 cytokines, all but TNF-β (not studied *in vivo*) were produced by leukocytes after antigen recall challenge in >4.5 month old infants immunized with BCG at birth (20, 34, 35, 39–55) (Supplementary Table 4).

**Figure 5:**
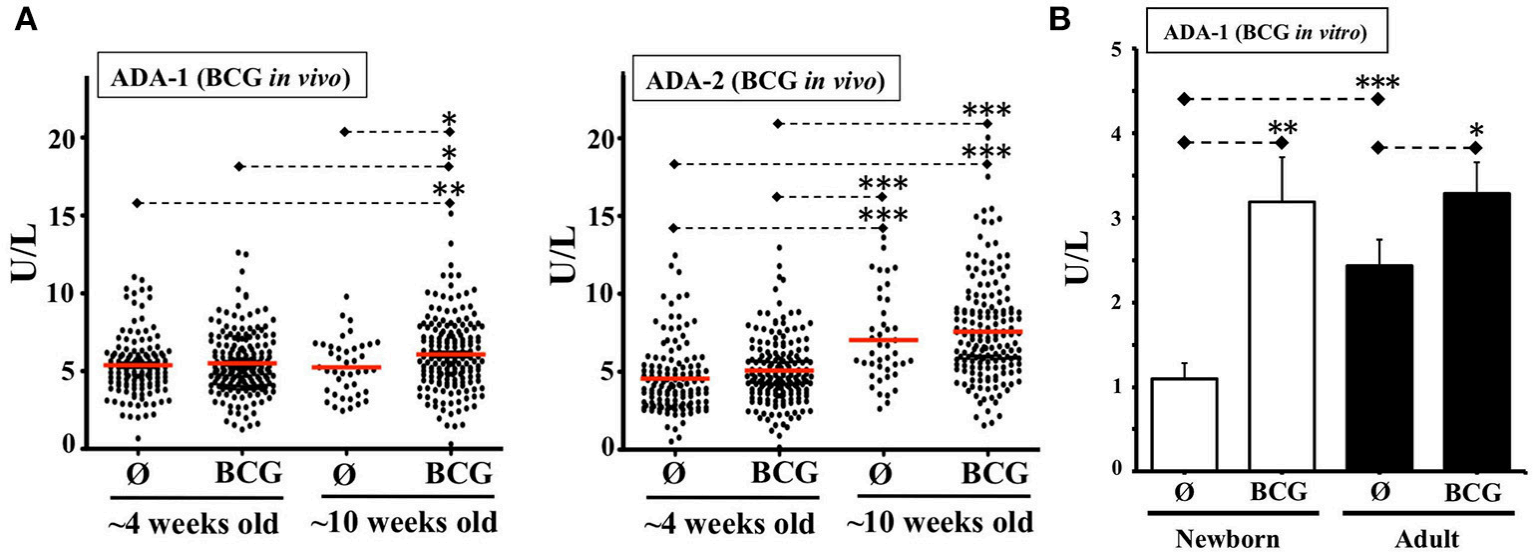
BCG vaccine induces plasma ADA activity *in vivo* and *in vitro*. **(A)** ADA-1 and ADA-2 activities in blood samples from a clinical study in Guinea-Bissau comparing birth-BCG (BCG) vs. delayed-BCG (Ø). Blood samples were obtained at ~4 and ~10 weeks of age (before immunization of delayed-BCG group). Each dot represents a study participant tested. The 4 weeks old infants group included 183 plasmas from early BCG and 131 from delayed-BCG group (Ø). The 10 weeks old infants group consisted of 169 plasmas from early BCG and 42 from delayed-BCG group (Ø). **(B)** ADA-1 activity in plasma from TCs immunized *in vitro* with BCG. Age-specific TCs were either stimulated with BCG (1:10v/v) or left unstimulated (Ø) and 48 h later plasmas were collected for ADA-1 activity testing (*N* = 10–13 participants per age group). U/L = Units per liter. *P*= * < 0.05; ** < 0.01; *** < 0.001.

Unlike newborn TNF responses to multiple other stimuli (e.g., Pam3) (24), TNF induction by Ag85A challenge was not inhibited by the relatively high adenosine (Ado) concentration of neonatal plasma. A possible explanation for this observation is that *Mycobacteria* spp. can induce enhanced expression of ADA, a biomarker enzyme that is elevated in TB infection (61), is critical for development and function of T cells, as evidenced by ADA-deficient SCID (35), and catabolizes Ado to the immunologically inert Inosine. To assess this mechanistic hypothesis and determine whether the NTC could model this relevant immune mechanism *in vitro*, we characterized ADA activity in the NTC. There are two forms of ADA, ADA-1, and ADA-2, the latter with little activity over physiological concentrations of Ado. Consistent with prior studies (62), newborn plasma demonstrated significantly lower ADA-1 activity than adult (Supplementary Figure 5). To enable ADA comparison of our *in vitro* NTC results with the effect of BCG on newborns *in vivo*, we measured ADA activity in blood plasma of 4 and 10 week-old infants from a randomized clinical trial among low-birth-weight infants in Guinea-Bissau (West Africa) (20) comparing BCG at birth immunization vs. a delayed BCG vaccination schedule (Figure 5A). ADA-2 increased with age in both groups, whereas ADA-1 increased from 4 to 10 weeks among BCG-vaccinated but not among controls. ADA-1 activity at 10 weeks old increased significantly in association to a birth dose of BCG. Since birth weight was strongly correlated with receipt of pentavalent or the subsequent BCG in the control group, a sensitivity analysis using linear regression was additionally performed. Controlling for birth weight did not change ADA results. In agreement with these results, and with the increased ADA activity induced by BCG on human adult MCs *in vitro* (63), a single dose of BCG induced a significant increase in ADA-1 activity in both the NTC and the ATC after 48 h *in vitro*, with a greater relative effect in newborns, such that BCG increased the relatively basal neonatal ADA-1 activity to a level similar to adults (Figure 5B).

### Autologous antigen-specific recall responses by age-specific DCs from HBV-immunized tissue constructs

**Figure 6:**
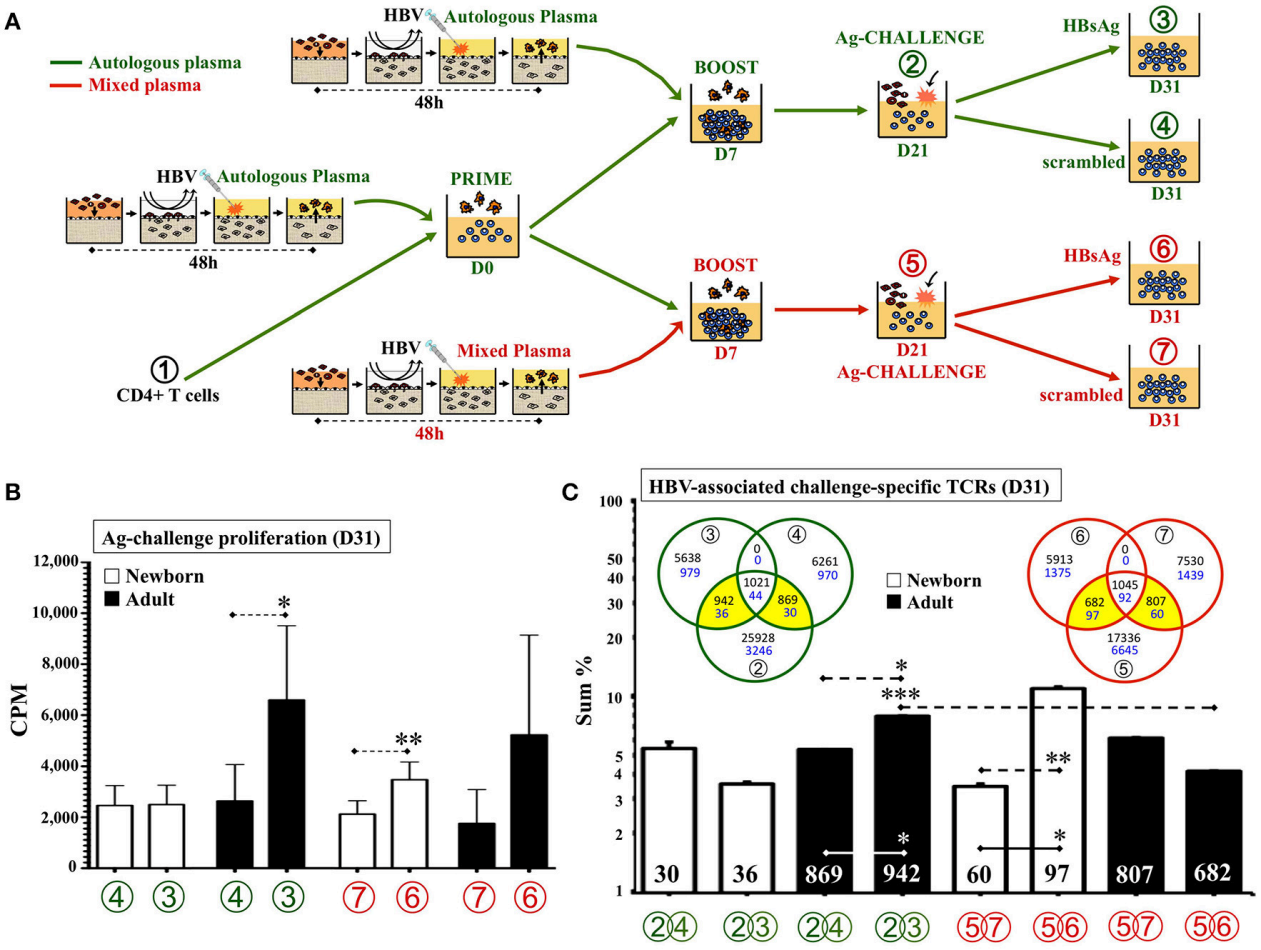
Human tissue constructs model autologous age- and antigen-specific responses after HBV immunization *in vitro*. **(A)** Assay design to assess autologous HBsAg-specific recall-responses after HBV newborn *in vitro* immunization. DCs from age-specific TCs stimulated with HBV (1:100v/v) were co-cultured (PRIME) with autologous CD4+ T cells ① for 7 days and then re-stimulated (BOOST) with a second round of autologous DCs form HBV-stimulated TCs (D7). At D21 of further culture (② and ⑤), resting T cells were collected, counted and split (5,000 cells/well) to undergo antigen challenge (Ag-CHALLENGE) for another 10 days (D31, ③,④, ⑥, and ⑦) using autologous monocytes and a peptide pool approach (HBsAg vs. scrambled sequence control). From D7 onward, half the experiments continue their culture under either 100% autologous plasma (green) or a 1:1v/v mix of newborn and adult plasmas (red). **(B)** Autologous newborn HBsAg-specific recall-proliferation induced by HBV *in vitro* immunization. Ag-challenge proliferation (CPM) was assessed at D31 for conditions ④ and (100% autologous plasma in green) ⑥ and ⑦ and (1:1v/v mixed plasma in red) on each age group according to assay design **(A)**, as described in methods. *N* = 10–15 participants/age group with ≥7 technical replicas/condition. **(C)** HBV-associated challenge-specific TCRs (D31). Cell samples from steps ①, ②, ③, ④, ⑤, ⑥, and ⑦ according to assay design **(A)** were taken for DNA extraction and high-throughput sequencing of the TCRβ CDR3 region, as described in Methods (*N* = 3/age-group). The sum of frequencies of productively rearranged non-shared TCRs from each peptide-challenge sample at D31 (③, ④, ⑥, and ⑦) that were also detected at corresponding D21 samples (② or ⑤), were plotted. The number of HBV-associated challenge-specific TCRs found (yellow areas of Venn diagrams) is indicated inside corresponding column. Blue numbers in Venn diagrams refer to newborns and black to adults. Green color refers to conditions using only 100% autologous plasma. Red color refers to conditions using mixed plasma from boost step onward. *P* = * < 0.05; ** < 0.01; *** < 0.001.

As HBV is a standard single-antigen alum-adjuvanted licensed vaccine containing a viral like particle of hepatitis B surface antigen (HBsAg) given at birth across the globe (64), we next modeled immunity to this vaccine *in vitro*. Considering that DCs from HBV-immunized TCs induced a modest but significant proliferation of newborn CD4 T cells at 1:100v/v dilution (Figure 3B) and that, a peptide pool approach successfully demonstrated BCG recall Ag-specific proliferative responses (Figure 4B), we followed a similar approach for antigen-challenge after HBV. Anticipating a positive memory response, we recruited HBV vaccinated adult donors. DCs from HBV-stimulated TCs were co-cultured with autologous total CD4+ T cells up to D15 before splitting cells and challenging them for an additional 10 days with autologous monocytes and either peptides spanning the entire protein sequence of HBV antigen HBsAg (22) or background control sequence-scrambled peptides (Supplementary Table 1 and Supplementary Figure 6). As noted *in vivo* after a single dose of HBV, with a substantial proportion of HBV non-responders and often negligible lymphoproliferation after infant HBsAg recall challenge (2, 56, 64–66), a single dose of vaccine resulted in insufficient detection of newborn HBsAg-associated recall proliferation above scrambled control. As effective HBV immunization *in vivo* typically requires >1 dose, we next implemented a TC boosting strategy *in vitro*. To provide this boosting HBV dose to already primed DC:T cell co-cultures, a second set of autologous TCs were *in vitro*-immunized with same HBV vaccine lot and dose (1:100v/v) and resulting autologous DCs were added to ongoing co-cultures at D7 (boost). Of note, second dose after a birth-dose of HBV is only recommended after passing 4-weeks of life (neonatal phase) (2, 64); by then, infant plasma is known to be distinct from that of newborns and adults (2, 28, 62). While *in vitro* HBV boosting would require using autologous infant (not neonatal) plasma from that point onward, from an *in vitro* modeling perspective there are challenging limitations in drawing the large volumes of autologous infant blood (>1 month of age) required for this step. Thus, to approximate infant plasma *in vitro*, we changed the natural relative presence of soluble immune-modulators halfway between neonatal and adult levels by mixing 1:1v/v the plasma of the newborn and the adult routinely tested side-by-side on each one of these assays. Accordingly, besides testing vaccine boosting *in vitro* with 100% neonatal plasma, we included a mixed plasma condition from the boost point onward (Figure 6A). Indeed, whereas no HBsAg-associated proliferation was detected above scrambled control (D31) when boosting under autologous newborn plasma, significant increase in HBsAg-specific proliferation was achieved when boosting under mixed plasma (Figure 6B). Measurement of antibodies against HBsAg (anti-HBsAg-Abs) in plasmas used for these HBV assays (Supplementary Table 2) demonstrated that mean anti-HBsAg-Ab titers were not significantly different between test conditions (Supplementary Figure 7), suggesting they did not account for the observed effect by mixed plasma.

To confirm our impression of the generation of HBsAg-specific responses *in vitro*, we characterized the HBsAg-specific T cell clones associated with HBV boosting under different plasma types. To this end, cells from three experiments (total *N* = 3 per age group) were taken at seven points throughout cultures (Figure 6A) and sent de-identified to a third-party laboratory for high-throughput sequencing of the CDR3 region using the ImmunoSEQ® assay (Adaptive Biotechnologies®; Seattle, WA). A pair-wise Morisita overlap index analysis for both, the nucleotide (NT) and predicted aminoacid (AA) sequences of detected TCRs of all samples (Supplementary Figure 8), indicated an acceptable level of independency (TCR clustering) between the six non-consanguineous participants with no overlap with the HUVEC control. Analysis of the number and frequency of detected productive rearranged TCR sequences, those likely reflecting single functional T cell, was focused on TCRs linked to HBV *in vitro* immunization (Figure 6C), as described in Methods. In contrast to the use of autologous plasma, analysis of the TCR repertoire resulting from NTCs boosted with HBV under 1:1v/v mixed plasma demonstrated a significant increase in number and frequency of HBsAg-associated TCR clones, as compared to scrambled control peptides. For ATCs the opposite pattern was observed, with mixed plasma during boosting significantly inhibiting expansion of HBsAg-specific clones.

## Discussion

Age-specific modeling of human vaccine responses *in vitro* may be crucial to accelerate and de-risk vaccine development as costly animal models reflect species-specific immunity and it is not feasible to conduct large scale human clinical trials in every target population for each potential candidate vaccine formulation including diverse combinations of vaccinal antigen, adjuvantation and formulation systems. In particular, modeling of vaccine-induced autologous naïve CD4+ T cell responses is important since T cell help to germinal center B cells is essential for durable vaccine-induced humoral immunity (67) and >90% of licensed U.S. vaccines are based on antigenic proteins, likely to initiate T cell responses *in vivo* (68). In this context, we report the first human *in vitro* system comprised of entirely human components that faithfully models age- and antigen-specific responses to licensed vaccines. Here, autonomously generated DCs from our age-specific microphysiologic human Tissue Constructs immunized *in vitro* with licensed pediatric vaccines, induced autologous naïve CD4+ T cell responses and single antigen-specific recall challenge responses, similar to those observed *in vivo*.

Our human TC model differs substantially from prior seemingly similar immunological *in vitro* approaches (10, 16–19) in its emphasis on: (1) immune ontogeny, including newborns who are at highest risk of infection and receive the greatest number of vaccines (69); (2) composition from exclusively endotoxin-free human biomaterials, providing a low background to enable detection of vaccine-induced antigen-specific responses; and (3) microphysiology, preserving autologous complex humoral and cellular signals that direct autonomous migration and differentiation of monocytes into DCs and their escape from tissues as age-specific antigen-presenting cells carrying vaccinal antigens, mirroring the efficient constitutive differentiation of monocytes into migratory DCs that occurs *in vivo* (11). Key to our culture approach is the use of relatively inexpensive autologous heparinized non-heated plasma that preserves age-specific factors that naturally shape innate and adaptive immune responses *in vivo* (31). Autonomous differentiation of primary monocytes into DCs occurred in autologous plasma with no exogenous cytokines or xenogenic additives, avoiding cellular differentiation to phenotypes that may not be relevant *in vivo* (70).

Providing a more natural microenvironment with minimal manipulation likely contributed to the ability of our human *in vitro* system to mirror *in vivo* vaccine responses, including: (1) innate DC maturation and migration in response to adjuvants and the self-adjuvanted BCG vaccine (71); (2) BCG-induced increase of plasma ADA activity in infants, a reported *in vivo* biomarker of infant BCG immunization (72) that we independently confirmed; (3) limited T lymphocyte proliferation in response to the T cell-independent pneumococcal polysaccharide vaccine (73); (4) vaccine-induced patterns of antigen-recall T cell cytokines, chemokines, and lymphoproliferation (2, 20, 34, 35, 37, 39–56, 64–66) (Supplementary Table 4); and (5) single-antigen responses with dosing and plasma requirements matching those needed to generate protective immunity *in vivo* [i.e., a single dose for BCG (23, 59) vs. multiple doses for HBV (64)]. Moreover, comparison of newborn and adult responses demonstrated that multiple aspects of our results also match age-specific differences noted in other human *in vitro* and *in vivo* studies (2, 24, 31, 32, 74), suggesting the validity of our approach: (i) greater and broader adult DC maturation and lymphoproliferation to a range of adjuvants and vaccines (69), with the notable exception of robust neonatal DC maturation by TLR7/8A, recently shown to be a highly effective neonatal vaccine adjuvant *in vivo* (71); (ii) increased responsiveness of newborn leukocytes in the presence of adult plasma, and lower responsiveness of adult cells cultured in newborn plasma (24); (iii) lower newborn plasma ADA activity baseline (62); (iv) higher adult cognate T cell responsiveness paralleling study participant vaccination history and immune status (74); and a narrower and Th1-polarized cytokine profile by BCG-immunized adults (75) as compared to a broader Th1/Th2 mixed profile by newborns *in vivo* (20, 34, 35, 39–55).

Few published studies have examined antigen presentation by human neonates *in vitro* (76–82), with only two demonstrating autologous naïve T cell responses toward a single-antigen (81, 82); neither of which used licensed newborn vaccines and both of which employed cytokines to differentiate monocytes into DCs, as well as exogenous additives to enforce T cell responses *in vitro*, approaches that may intrinsically compromise the physiologic relevance of the findings and their *in vivo* translation. Moreover, to our knowledge our *in vitro* platform is the first culture system that achieved the induction of human newborn autologous naïve antigen-specific CD4+ T lymphocyte responses to licensed vaccines *in vitro* matching responses observed *in vivo*.

Overall, our results strongly suggest that microphysiologic *in vitro* cultures can model both innate and adaptive autologous age-specific human immune responses to adjuvants and licensed vaccines. Our culture approach demonstrates advantages over clinical trials as it compares the same study participant for both control and test conditions, thereby enhancing statistical power by limiting unwanted genetic and epigenetic variability intrinsic to human clinical trials, and enables large-scale human studies of combinations of candidate vaccinal antigens, adjuvantation systems and formulations, which is not feasible *in vivo* given the costs of conducting large-scale human clinical trials. As with any new technology, our culture model also has limitations such as the lack of physiologic fluid flow, natural self-driven DC:T cell interactions (as fixed DC:T cell ratios were used for proof of concept comparisons) and naïve B cell Ab responses, a common vaccine correlate of protection (68). Future studies should explore use of whole blood and interstitial flow (microfluidics) enabling natural transit of migratory cells to reflect the complexity of cell migration and cell-cell interactions inherent to tissues *in vivo*. Modeling lymphoid cell heterogeneity will likely be necessary to generate autologous functional germinal centers and Ab production; a technical aspect that thus far has apparently only been demonstrated under markedly non-physiologic conditions (83).

In sum, our microphysiologic culture system enables autonomous generation of human DCs that can stimulate autologous naïve CD4 T cells to replicate age-specific single-antigen immune responses to licensed vaccines, as noted *in vivo*. Our newborn and adult culture systems offer practical and technical advantages over current systems and in principle can be used to model responses to additional vulnerable populations such as the frail, elderly and chronically ill. Our approach offers a unique platform for human pre-clinical immunological studies informing age-targeted vaccine effectiveness *in vitro* to accelerate, enhance, and de-risk vaccine development, including design of single-dose early life vaccines, that can increase global vaccine coverage, reduce infant vulnerability, and provide major public health benefits (2).

## Data availability statement

The raw data supporting the conclusions of this manuscript will be made available by the authors, without undue reservation, to any qualified researcher. Some data will be deposited to NIAID's *ImmPort* website.

## Author contributions

GS-S conceived, designed and executed the experiments, analyzed the data, and wrote the manuscript. PF, CS, IB, and GM assisted with execution of experiments and provided manuscript edits. KS-A analyzed TCR data. DH assisted with TCR data analysis and provided manuscript edits. KJ and CB designed and recruited the study cohort in Guinea-Bissau (20) whose plasma samples were studied and assisted with ADA data analyses. OL provided key intellectual input, funding, and infrastructure support to this project, and edited the manuscript.

### Conflict of interest statement

Dr. Levy's Lab has received sponsored research support from 3M Drug Delivery Systems, MedImmune, and Crucell (Johnson & Johnson). GS-S, CS, and OL are named on patent application entitled Tissue Constructs and Uses Thereof (United States Application Serial No. 14/383,358) filed by Boston Children's Hospital. DH is an employee of Adaptive Biotechnologies. The remaining authors declare that the research was conducted in the absence of any commercial or financial relationships that could be construed as a potential conflict of interest.

## References

[1] Kollmann TR Levy O Montgomery RR Goriely S. Innate immune function by Toll-like receptors: distinct responses in newborns and the elderly. Immunity (2012) 37:771–83. 10.1016/j.immuni.2012.10.014 23159225 PMC3538030

[2] Sanchez-Schmitz G Levy O. Development of newborn and infant vaccines. Sci Transl Med. (2011) 3:90ps27. 10.1126/scitranslmed.3001880 21734174 PMC4108897

[3] Pronker ES Weenen TC Commandeur H Claassen EH Osterhaus AD. Risk in vaccine research and development quantified. PLoS ONE (2013) 8:e57755. 10.1371/journal.pone.0057755 23526951 PMC3603987

[4] Leist M Hartung T. Inflammatory findings on species extrapolations: humans are definitely no 70-kg mice. Arch Toxicol. (2013) 87:563–7. 10.1007/s00204-013-1038-0 23503654 PMC3604596

[5] Vaccari M Franchini G. T cell subsets in the germinal center: lessons from the macaque model. Front Immunol. (2018) 9:348. 10.3389/fimmu.2018.00348 29535724 PMC5834428

[6] Kambayashi T Laufer TM. Atypical MHC class II-expressing antigen-presenting cells: can anything replace a dendritic cell? Nat Rev Immunol. (2014) 14:719–30. 10.1038/nri3754 25324123

[7] Benvenuti F. The dendritic cell synapse: a life dedicated to T cell activation. Front Immunol. (2016) 7:70. 10.3389/fimmu.2016.00070 27014259 PMC4780025

[8] Tapia-Calle G Stoel M de Vries-Idema J Huckriede A. Distinctive responses in an *in vitro* human dendritic cell-based system upon stimulation with different influenza vaccine formulations. Vaccines (2017) 5:E21. 10.3390/vaccines5030021 28792466 PMC5620552

[9] Randolph GJ Ochando J Partida-Sanchez S. Migration of dendritic cell subsets and their precursors. Annu Rev Immunol. (2008) 26:293–316. 10.1146/annurev.immunol.26.021607.090254 18045026

[10] Randolph GJ Beaulieu S Lebecque S Steinman RM Muller WA. Differentiation of monocytes into dendritic cells in a model of transendothelial trafficking. Science (1998) 282:480–3. 10.1126/science.282.5388.480 9774276

[11] Randolph GJ Inaba K Robbiani DF Steinman RM Muller WA. Differentiation of phagocytic monocytes into lymph node dendritic cells *in vivo*. Immunity (1999) 11:753–61. 10.1016/S1074-7613(00)80149-1 10626897

[12] Randolph GJ Sanchez-Schmitz G Angeli V Randolph GJ Sanchez-Schmitz G Angeli V. Factors and signals that govern the migration of dendritic cells via lymphatics: recent advances. Springer Semin Immunopathol. (2005) 26:273–87. 10.1007/s00281-004-0168-0 15338191

[13] Randolph GJ Beaulieu S Pope M Sugawara I Hoffman L Steinman RM. A physiologic function for p-glycoprotein (MDR-1) during the migration of dendritic cells from skin via afferent lymphatic vessels. Proc Natl Acad Sci USA. (1998) 95:6924–9. 10.1073/pnas.95.12.6924 9618515 PMC22688

[14] Flores-Romo L. *In vivo* maturation and migration of dendritic cells. Immunology (2001) 102:255–62. 10.1046/j.1365-2567.2001.01204.x 11298823 PMC1783189

[15] Townsley MI. Structure and composition of pulmonary arteries, capillaries, and veins. Compr Physiol. (2012) 2:675–709. 10.1002/cphy.c100081 23606929 PMC3630377

[16] Randolph GJ Sanchez-Schmitz G Liebman RM Schäkel K. The CD16^+^ (FcγRIII^+^) subset of human monocytes preferentially becomes migratory dendritic cells in a model tissue setting. J Exp Med. (2002) 196:517–27. 12186843 10.1084/jem.20011608 PMC2196052

[17] Qu C Moran TM Randolph GJ. Autocrine type I IFN and contact with endothelium promote the presentation of influenza A virus by monocyte-derived. APJ Immunol C (2003) 170:1010–8. 10.4049/jimmunol.170.2.1010 12517968

[18] Schanen BC Drake DR III. A novel approach for the generation of human dendritic cells from blood monocytes in the absence of exogenous factors. J Immunol Methods (2008) 335:53–64. 10.1016/j.jim.2008.02.021 18423481

[19] Higbee RG Byers AM Dhir V Drake D Fahlenkamp HG Gangur J. An immunologic model for rapid vaccine assessment – a clinical trial in a test tube. Altern Lab Anim. (2009) 37 (Suppl. 1):19–27. 19807200 10.1177/026119290903701S05

[20] Jensen KJ Larsen N Biering-Sørensen S Andersen A Eriksen HB Monteiro I. Heterologous immunological effects of early BCG vaccination in low-birth-weight infants in Guinea-Bissau: a randomized-controlled trial. J Infect Dis. (2015) 211:956–67. 10.1093/infdis/jiu508 25210141 PMC4340366

[21] Huygen K Lozes E Gilles B Drowart A Palfliet K Jurion F. Mapping of TH1 helper T-cell epitopes on major secreted mycobacterial antigen 85A in mice infected with live *Mycobacterium bovis*. BC Infect Immun. (1994) 62:363–70. 7507889 10.1128/iai.62.2.363-370.1994 PMC186116

[22] Desombere I Gijbels Y Verwulgen A Leroux-Roels G. Characterization of the T cell recognition of hepatitis B surface antigen (HBsAg) by good and poor responders to hepatitis B vaccines. Clin Exp Immunol. (2000) 122:390–9. 10.1046/j.1365-2249.2000.01383.x 11122245 PMC1905794

[23] Merck & Co I. PNEUMOVAX® 23 (pneumococcal vaccine polyvalent). Prescribing information. (2016).

[24] Levy O Coughlin M Cronstein BN Roy RM Desai A Wessels MR. The adenosine system selectively inhibits TLR-mediated TNF-alpha production in the human newborn. J Immunol. (2006) 177:1956–66. 10.4049/jimmunol.177.3.1956 16849509 PMC2881468

[25] van Montfoort N van der Aa E van den Bosch A Brouwers H Vanwolleghem T Janssen HLA. Hepatitis B virus surface antigen activates myeloid dendritic cells via a soluble CD14-dependent mechanism. J Virol. (2016) 90:6187–99. 10.1128/JVI.02903-15 27099316 PMC4936135

[26] Ben Haij N Planès R Leghmari K Serrero M Delobel P Izopet J. HIV-1 tat protein induces production of proinflammatory cytokines by human dendritic cells and monocytes/macrophages through engagement of TLR4-MD2-CD14 complex and activation of NF-kappaB pathway. PLoS ONE (2015) 10:e0129425. 10.1371/journal.pone.0129425 26090662 PMC4474861

[27] Sánchez-Torres C García-Romo GS Cornejo-Cortés MA Rivas-Carvalho A Sánchez-Schmitz G.Sanchez-Torres C, et al. CD16^+^ and CD16^−^ human blood monocyte subsets differentiate *in vitro* to dendritic cells with different abilities to stimulate CD4^+^ T cells. Int Immunol. (2001) 13:1571–81. 10.1093/intimm/13.12.1571 11717198

[28] PrabhuDas M Adkins B Gans H King C Levy O Ramilo O. Challenges in infant immunity: implications for responses to infection and vaccines. Nat Immunol. (2011) 12:189–94. 10.1038/ni0311-189 21321588

[29] Kumari M Saxena RK. Relative efficacy of uptake and presentation of *Mycobacterium bovis* BCG antigens by type I mouse lung epithelial cells and peritoneal macrophages. Infect Immun. (2011) 79:3159–67. 10.1128/IAI.05406-11 21646448 PMC3147596

[30] Carlson CS Emerson RO Sherwood AM Desmarais C Chung MW Parsons JM. Using synthetic templates to design an unbiased multiplex PCR assay. Nat Commun. (2013) 4:2680. 10.1038/ncomms3680 24157944

[31] Pettengill MA van Haren SD Levy O. Soluble mediators regulating immunity in early life. Front Immunol. (2014) 5:457. 10.3389/fimmu.2014.00457 25309541 PMC4173950

[32] Levy O Suter EE Miller RL Wessels MR. Unique efficacy of Toll-like receptor 8 agonists in activating human neonatal antigen-presenting cells. Blood (2006) 108:1284–90. 10.1182/blood-2005-12-4821 16638933 PMC1895876

[33] Potolicchio I Chitta S Xu X Fonseca D Crisi G Horejsi V Conformational variation of surface class II MHC proteins during myeloid dendritic cell differentiation accompanies structural changes in lysosomal MII. J Immunol C (2005) 175:4935–47. 10.4049/jimmunol.175.8.4935 16210595

[34] Casanova JL Abel L. Genetic dissection of immunity to mycobacteria: the human model. Annu Rev Immunol. (2002) 20:581–620. 10.1146/annurev.immunol.20.081501.125851 11861613

[35] Pachlopnik Schmid J Güngör T Seger R. Modern management of primary T-cell immunodeficiencies. Pediatr Allergy Immunol. (2014) 25:300–13. 24383740 10.1111/pai.12179

[36] Teschner D Distler E Wehler D Frey M Marandiuc D Langeveld K. Depletion of naive T cells using clinical grade magnetic CD45RA beads: a new approach for GVHD prophylaxis. Bone Marrow Transplant. (2014) 49:138–44. 10.1038/bmt.2013.114 23933765

[37] van den Biggelaar AH Pomat W Bosco A Phuanukoonnon S Devitt CJ Nadal-Sims MA. Pneumococcal conjugate vaccination at birth in a high-risk setting: no evidence for neonatal T-cell tolerance. Vaccine (2011) 29:5414–20. 10.1016/j.vaccine.2011.05.065 21645573 PMC3146700

[38] Thurley K Gerecht D Friedmann E Höfer T. Three-dimensional gradients of cytokine signaling between T Cells. PLoS Comput Biol. (2015) 11:e1004206. 10.1371/journal.pcbi.1004206 25923703 PMC4414419

[39] Murray RA Mansoor N Harbacheuski R Soler J Davids V Soares A. Bacillus calmette guerin vaccination of human newborns induces a specific, functional CD8+ T cell response. J Immunol. (2006) 177:5647–51. 10.4049/jimmunol.177.8.5647 17015753

[40] Randhawa AK Shey MS Keyser A Peixoto B Wells RD de Kock M. Association of human TLR1 and TLR6 deficiency with altered immune responses to BCG vaccination in South African infants. PLoS Pathog. (2011) 7:e1002174. 10.1371/journal.ppat.1002174 21852947 PMC3154845

[41] Djuardi Y Sartono E Wibowo H Supali T Yazdanbakhsh M. A longitudinal study of BCG vaccination in early childhood: the development of innate and adaptive immune responses. PLoS ONE (2010) 5:e14066. 10.1371/journal.pone.0014066 21124909 PMC2988815

[42] Sartono E Lisse IM Terveer EM van de Sande PJ Whittle H Fisker AB. Oral polio vaccine influences the immune response to BCG vaccination. A natural experiment. PLoS ONE (2010) 5:e10328. 10.1371/journal.pone.0010328 20502641 PMC2873948

[43] Burl S Adetifa UJ Cox M Touray E Ota MO Marchant A. Delaying bacillus calmette-guerin vaccination from birth to 4 1/2 months of age reduces postvaccination Th1 and IL-17 responses but leads to comparable mycobacterial responses at 9 months of age. J Immunol. (2010) 185:2620–8. 10.4049/jimmunol.1000552 20644160

[44] Akkoc T Aydogan M Yildiz A Karakoc-Aydiner E Eifan A Keles S. Neonatal BCG vaccination induces IL-10 production by CD4^+^ CD25^+^ T cells. Pediatr Allergy Immunol. (2010) 21:1059–63. 10.1111/j.1399-3038.2010.01051.x 20977501

[45] Kagina BM Abel B Bowmaker M Scriba TJ Gelderbloem S Smit E. Delaying BCG vaccination from birth to 10 weeks of age may result in an enhanced memory CD4 T cell response. Vaccine (2009) 27:5488–95. 10.1016/j.vaccine.2009.06.103 19616494 PMC2745558

[46] Finan C Ota MO Marchant A Newport MJ. Natural variation in immune responses to neonatal *Mycobacterium bovis* Bacillus Calmette-Guerin (BCG) Vaccination in a Cohort of Gambian infants. PLoS ONE (2008) 3:e3485. 10.1371/journal.pone.0003485 18941532 PMC2567029

[47] Matee M Lahey T Vuola JM Mtei L Cole BF Bakari M. Baseline mycobacterial immune responses in HIV-infected adults primed with bacille Calmette-Guerin during childhood and entering a tuberculosis booster vaccine trial. J Infect Dis. (2007) 195:118–23. 10.1086/509896 17152015 PMC2871300

[48] Watkins ML Semple PL Abel B Hanekom WA Kaplan G Ress SR. Exposure of cord blood to *Mycobacterium bovis* BCG induces an innate response but not a T-cell cytokine response. Clin Vaccine Immunol. (2008) 15:1666–73. 10.1128/CVI.00202-08 18815231 PMC2583525

[49] Vekemans J Amedei A Ota MO D'Elios MM Goetghebuer T Ismaili J. Neonatal bacillus Calmette-Guerin vaccination induces adult-like IFN-gamma production by CD4+ T lymphocytes. Eur J Immunol. (2001) 31:1531–5. 10.1002/1521-4141(200105)31:5<1531::AID-IMMU1531>3.0.CO;2-1 11465110

[50] Soares AP Scriba TJ Joseph S Harbacheuski R Murray RA Gelderbloem SJ. Bacillus Calmette-Guerin vaccination of human newborns induces T cells with complex cytokine and phenotypic profiles. J Immunol. (2008) 180:3569–77. 10.4049/jimmunol.180.5.3569 18292584 PMC2842001

[51] Marchant A Goetghebuer T Ota MO Wolfe I Ceesay SJ De Groote D. Newborns develop a Th1-type immune response to *Mycobacterium bovis* bacillus Calmette-Guerin vaccination. J Immunol. (1999) 163:2249–55. 10438968

[52] Jones BE Young SM Antoniskis D Davidson PT Kramer F Barnes PF. Relationship of the manifestations of tuberculosis to CD4 cell counts in patients with human immunodeficiency virus infection. Am Rev Respir Dis. (1993) 148:1292–7. 10.1164/ajrccm/148.5.1292 7902049

[53] Oni T Gideon HP Bangani N Tsekela R Seldon R Wood K. Smoking BCG, and employment and the risk of tuberculosis infection in HIV-infected persons in South Africa. PLoS ONE (2012) 7:e47072. 10.1371/journal.pone.0047072 23056584 PMC3467259

[54] Oni T Gideon HP Bangani N Tsekela R Seldon R Wood K. Risk factors associated with indeterminate gamma interferon responses in the assessment of latent tuberculosis infection in a high-incidence environment. Clin Vaccine Immunol. (2012) 19:1243–7. 10.1128/CVI.00166-12 22718129 PMC3416070

[55] Arikan C Bahceciler NN Deniz G Akdis M Akkoc T Akdis CA. Bacillus Calmette-Guerin-induced interleukin-12 did not additionally improve clinical and immunologic parameters in asthmatic children treated with sublingual immunotherapy. Clin Exp Allergy (2004) 34:398–405. 10.1111/j.1365-2222.2004.01869.x 15005733

[56] Ota MO Vekemans J Schlegel-Haueter SE Fielding K Whittle H Lambert PH. Hepatitis B immunisation induces higher antibody and memory Th2 responses in new-borns than in adults. Vaccine (2004) 22:511–9. 10.1016/j.vaccine.2003.07.020 14670334

[57] Roth A Glaesener S Schütz K Meyer-Bahlburg A. Reduced number of transitional and naive B cells in addition to decreased BAFF levels in response to the T cell independent immunogen pneumovax(R)23. PLoS ONE (2016) 11:e0152215. 10.1371/journal.pone.0152215 27031098 PMC4816312

[58] Wyeth Pharmaceuticals I Pneumococcal 13-valent Conjugate Vaccine. Package insert. (2016).

[59] Share JB Description of BCG Vaccine SSI. Institut SS (2011).

[60] Huygen K. The Immunodominant T-cell epitopes of the mycolyl-transferases of the antigen 85 complex of *M. tuberculosis*. Front Immunol. (2014) 5:321. 10.3389/fimmu.2014.00321 25071781 PMC4089088

[61] Dinnes J Deeks J Kunst H Gibson A Cummins E Waugh N. A systematic review of rapid diagnostic tests for the detection of tuberculosis infection. Health Technol Assess. (2007) 11:1–196. 10.3310/hta11030 17266837

[62] Pettengill M Robson S Tresenriter M Millán JL Usheva A Bingham T. Soluble ecto-5'-nucleotidase (5'-NT), alkaline phosphatase, and adenosine deaminase (ADA1) activities in neonatal blood favor elevated extracellular adenosine. J Biol Chem. (2013) 288:27315–26. 10.1074/jbc.M113.484212 23897810 PMC3779727

[63] Kashyap RS Husain AA Morey SH Panchbhai MS Deshpande PS Purohit HJ. Assessment of immune response to repeat stimulation with BCG vaccine using *in vitro* PBMC model. J Immune Based Ther Vaccines (2010) 8:3. 10.1186/1476-8518-8-3 20509931 PMC2890520

[64] Merck & Co I Package Insert for RECOMBIVAX HB. (2016).

[65] Avanzini MA Belloni C Soncini R Ciardelli L de Silvestri A Pistorio A. Increment of recombinant hepatitis B surface antigen-specific T-cell precursors after revaccination of slow responder children. Vaccine (2001) 19:2819–24. 10.1016/S0264-410X(01)00007-X 11282192

[66] Nyström J Cardell K Björnsdottir TB Fryden A Hultgren C Sällberg M. Improved cell mediated immune responses after successful re-vaccination of non-responders to the hepatitis B virus surface antigen (HBsAg) vaccine using the combined hepatitis A and B vaccine. Vaccine (2008) 26:5967–72. 10.1016/j.vaccine.2008.08.054 18804140

[67] Tangye SG Ma CS Brink R Deenick EK. The good, the bad and the ugly - TFH cells in human health and disease. Nat Rev Immunol. (2013) 13:412–26. 10.1038/nri3447 23681096

[68] Administration, U.S.F.a.D Vaccines Licensed for Use in the United States. (2018). Available from: http://www.fda.gov/BiologicsBloodVaccines/Vaccines/ApprovedProducts/ucm093833.htm (Accessed October 11, 2016).

[69] Kollmann TR Kampmann B Mazmanian SK Marchant A Levy O. Protecting the newborn and young infant from infectious diseases: lessons from immune ontogeny. Immunity (2017) 46:350–63. 10.1016/j.immuni.2017.03.009 28329702

[70] Guo X Zhou Y Wu T Zhu X Lai W Wu L. Generation of mouse and human dendritic cells *in vitro*. J Immunol Methods (2016) 432:24–9. 10.1016/j.jim.2016.02.011 26876301

[71] Dowling DJ Scott EA Scheid A Bergelson I Joshi S Pietrasanta C. Toll-like receptor 8 agonist nanoparticles mimic immunomodulating effects of the live BCG vaccine and enhance neonatal innate and adaptive immune responses. J Allergy Clin Immunol. (2017) 140:1339–50. 10.1016/j.jaci.2016.12.985 28343701 PMC5667586

[72] Thora S Rajsekaran P Chhaparwal BC. Serum adenosine deaminase estimation in relation to BCG vaccination. Indian Pediatr. (1995) 32:1087–8. 8984046

[73] Karasartova D Gazi U Tosun O Gureser AS Sahiner IT Dolapci M. Anti-pneumococcal vaccine-induced cellular immune responses in post-traumatic splenectomized individuals. J Clin Immunol. (2017) 37:388–96. 10.1007/s10875-017-0397-3 28488145

[74] Kowalewicz-Kulbat M Szpakowski P Locht C Biet F Kaplonek P Krawczyk KT. Tuberculin skin test reaction is related to memory, but not naive CD4(+) T cell responses to mycobacterial stimuli in BCG-vaccinated young adults. Vaccine (2018) 36:4566–77. 10.1016/j.vaccine.2018.05.068 29909133

[75] Hoft DF Kemp EB Marinaro M Cruz O Kiyono H McGhee JR. A double-blind, placebo-controlled study of *Mycobacterium*-specific human immune responses induced by intradermal bacille Calmette-Guerin vaccination. J Lab Clin Med. (1999) 134:244–52. 10.1016/S0022-2143(99)90204-4 10482309

[76] Tonon S Goriely S Aksoy E Pradier O Del Giudice G Trannoy E. Bordetella pertussis toxin induces the release of inflammatory cytokines and dendritic cell activation in whole blood: impaired responses in human newborns. Eur J Immunol. (2002) 32:3118–25. 10.1002/1521-4141(200211)32:11<3118::AID-IMMU3118>3.0.CO;2-B 12385032

[77] Goriely S Van Lint C Dadkhah R Libin M De Wit D Demonté D. A defect in nucleosome remodeling prevents IL-12(p35) gene transcription in neonatal dendritic cells. J Exp Med. (2004) 199:1011–6. 10.1084/jem.20031272 15051764 PMC2211877

[78] Philbin VJ Dowling DJ Gallington LC Cortés G Tan Z Suter EE. Imidazoquinoline Toll-like receptor 8 agonists activate human newborn monocytes and dendritic cells through adenosine-refractory and caspase-1-dependent pathways. J Allergy Clin Immunol. (2012) 130:195–204 e9. 10.1016/j.jaci.2012.02.042 22521247 PMC3387351

[79] Liu EM Law HK Lau YL. *Mycobacterium bovis* bacillus Calmette-Guerin treated human cord blood monocyte-derived dendritic cells polarize naive T cells into a tolerogenic phenotype in newborns. World J Pediatr. (2010) 6:132–40. 10.1007/s12519-010-0019-0 20127220

[80] Li G Kim YJ Broxmeyer HE. Macrophage colony-stimulating factor drives cord blood monocyte differentiation into IL-10(high)IL-12absent dendritic cells with tolerogenic potential. J Immunol (2005) 174:4706–17. 10.4049/jimmunol.174.8.4706 15814695

[81] Matthews NC Power UF Reen DJ. Neonatal human autologous dendritic cells pulsed with recombinant protein antigen prime the generation of non-polarized CD4 T-cell effectors. Int Immunol (2007) 19:703–12. 10.1093/intimm/dxm025 17493958

[82] Safdar A Decker WK Li S Xing D Robinson SN Yang H. De novo T-lymphocyte responses against baculovirus-derived recombinant influenzavirus hemagglutinin generated by a naive umbilical cord blood model of dendritic cell vaccination. Vaccine (2009) 27:1479–84. 10.1016/j.vaccine.2009.01.017 19185049

[83] Dinnis DM James DC. Engineering mammalian cell factories for improved recombinant monoclonal antibody production: lessons from nature? Biotechnol Bioeng. (2005) 91:180–9. 10.1002/bit.20499 15880827

