# Supplementary material for: Microphysiologic Human Tissue Constructs Reproduce Autologous Age-Specific BCG and HBV Primary Immunization in vitro
Source: Front Immunol. 2018 Nov 20;9:2634. doi: 10.3389/fimmu.2018.02634 (PMC6256288; doi:10.3389/fimmu.2018.02634)
Supplement: Supplementary file 7 [file Data_Sheet_1.docx]

Supplementary Material

**Microphysiologic human tissue constructs reproduce age-specific BCG and HBV primary immunization *in vitro***

**Guzman Sanchez-Schmitz****^*^, Chad R. Stevens, Ian A. Bettencourt, Peter Flynn, Klaus Schmitz-Abe, Gil Metser, David Hamm, Kristoffer J. Jensen, Christine Benn, Ofer Levy^*^**

*** Correspondence:**

**Guzman Sanchez-Schmitz, M.Sc., Ph.D.**

guzman.sanchez-schmitz@childrens.harvard.edu; guzmanss@hotmail.com

**Ofer Levy**

ofer.levy@childrens.harvard.edu

# 1. Supplementary Tables

**Supplementary Table 1. Aminoacid sequences of 50%-overlapping synthetic peptides used for antigen challenge studies.** Ag85A is a protein found in BCG vaccine and Mycobacterium tuberculosis; HBsAg is a surface protein of Hepatitis B virus and the only targeted antigen in HBV vaccine.

|  | Ag85A [1]  (BCG vaccine and Mycobacterium tuberculosis) | | HBsAg [2]  (HBV vaccine and Hepatitis B virus) | |
| --- | --- | --- | --- | --- |
|  | **Ag85A** | **Scrambled** | **HBsAg** | **Scrambled** |
| 1 | FSRPGLPVEYLQVPSPSMGR | GEPVSQSPMSPFRLLPYVGR | MENITSGFLG | SNTELFMGIG |
| 2 | LQVPSPSMGRDIKVQFQSGG | GPRSQPIMGSQVVFKSDLQG | SGFLGPLLVLQAGFFLLTR | LGPFLFQLRLSALFGLGTV |
| 3 | DIKVQFQSGGANSPALYLLD | NAIQVSDLGKFDPQAGLSLY | FFLLTRILTIPQSLD | LFTQLPLRLSTIDFI |
| 4 | ANSPALYLLDGLRAQDDFSG | DALAGNLSPQDFLDAGLSYR | TRILTIPQSLDSWWTSLNF | PSNWLSLILRWISTFQTDT |
| 5 | GLRAQDDFSGWDINTPAFEW | GPWIFLDAWFSDQRATDEGN | TSLNFLGGSPVC | NLSGSGVPCLTF |
| 6 | WDINTPAFEWYDQSGLSVVM | LDWTMEDAVSNWGVISPYFQ | NSQSPTSNHSPTSCPPICP | QPSSHCNPSPCSIPNTPST |
| 7 | YDQSGLSVVMPVGGQSSFYS | LSPMSGYQDFGVYSSVSVGQ | PICPGYRWMCLRRFIIFL | CRIRYFGIFCILMPWRPL |
| 8 | PVGGQSSFYSDWYQPACGKA | YKAVGSDYFWPGPCQQSASG | GMLPVCPLIPGSTTTNTG | PNLLTSGCTMPTPGITVG |
| 9 | DWYQPACGKAGCQTYKWETF | TWKATGQGKFACCWDQYPEY | TTNTGPCKTCTTPAQG | TPGTATTKCNCPGTQT |
| 10 | GCQTYKWETFLTSELPGWLQ | TEQGTEGLLPLWCSYTQWFK | PTDGNCTCIPIPSSWAFA | PAICDSPWTIFPASTGCN |
| 11 | LTSELPGWLQANRHVKPTGS | KTLGEGPAWSTNLPRHVLQS | SVRFSWLSLLVPFVQWF | RWSFVLFQSWVVLSFPL |
| 12 | ANRHVKPTGSAVVGLSMAAS | NVRAPAVLGHSVATKSMAGS | VPFVQWFVGLSPTVWLSA | PFWAVSQLGSTPWVLVVF |
| 13 | AVVGLSMAASSALTLAIYHP | AHLVAYPAMTSALSAGVLSI | SPTVWLSAIWMMWYWGPS | GWPWSWAMYVMPLISSTW |
| 14 | SALTLAIYHPQQFVYAGAMS | AQVGLTAPAQHYASYMFSIL | YWGPSLYSIVSPFIPL | PFSWPILSVPYLGYIS |
| 15 | QQFVYAGAMSGLLDPSQAMG | GFGMAQLQYQAPSSDMVGAL | SPFIPLLPIFFCLWVYI | PWPSFYPCILFLIVFLI |
| 16 | GLLDPSQAMGPTLIGLAMGD | GMDLGPGIDMTLSLPALAGQ |  |  |
| 17 | PTLIGLAMGDAGGYKASDMW | GLTGWGSMDIAADPKLYMGA |  |  |
| 18 | AGGYKASDMWGPKEDPAWQR | APGDSPWAKGMGARQYWKDE |  |  |
| 19 | GPKEDPAWQRNDPLLNVGKL | KAPQEPWDLGVLKPGNDNLR |  |  |
| 20 | NDPLLNVGKLIANNTRVWVY | PTVAILDNKNWLVVNRYNGL |  |  |
| 21 | IANNTRVWVYCGNGKPSDLG | AIGLCVVGWDPKYNGNSRTN |  |  |
| 22 | CGNGKPSDLGGNNLPAKFLE | GPLNGLNDEPKFAGCLKGSN |  |  |
| 23 | GNNLPAKFLEGFVRTSNIKF | LNFEGAILNSFRPKKFNVGT |  |  |
| 24 | GFVRTSNIKFQDAYNAGGGH | ADGSHGAIGYKQTVNFGRFN |  |  |
| 25 | QDAYNAGGGHNGVFDFPDSG | GDPAVGFGADNGQGDHFNYS |  |  |
| 26 | NGVFDFPDSGTHSWEYWGAQ | WTGYDGHSEADFNPWVFSGQ |  |  |
| 27 | THSWEYWGAQLNAMKPDLQR | EWLWMRSAGPQLNYQHKTAD |  |  |
| 28 | MKPDLQRALGATPNTGPAPQGA | PLAAQLPRGTMPGTNPGDQAAK |  |  |

**Supplementary Table 2. Antibody titers against Hepatitis B surface Antigen found in non-heated plasmas used for HBV antigen challenge studies.** Measurements were done by The Department of Laboratory Medicine of Boston Children’s Hospital. Titers of mixed plasmas were not measured but inferred from mixing 1:1v/v the newborn and adult plasmas. ID = donor identification. mIU/mL = mili-International Units per mL.

| **Newborn plasma (ID)** | **mIU/mL** | **Adult plasma (ID)** | **mIU/mL** | **50%v/v Mixed plasma** |
| --- | --- | --- | --- | --- |
|  |  | A1296-a67 | **22.22** |  |
|  |  | A1378-a68 | **2175.66** |  |
| C1179-a69 | **3.07** | A1383-a69 | **60.37** | **31.72** |
| C1162-a73 | **6985.19** | A1390-a73 | **768.98** | **3877.085** |
| C1191-a76 | **379.99** | A1404-a76 | **9993.75** | **5186.87** |
| C1076-a78 | **19.89** | A1408-a78 | **175.29** | **97.59** |
| C1124-a79 | **64.45** | A1415-a79 | **690.05** | **377.25** |
| C1194-a80 | **891.41** | A1416-a80 | **1055.55** | **973.48** |
| C1243-a82 | **0.001** | A1456-a82 | **0.82** | **0.41** |
| C1246-a84 | **6.74** | A1441-a84 | **0.92** | **3.83** |
| C1259-a85 | **9741.48** | A1470-a85 | **0.74** | **4871.11** |
| C1229-a86 | **1788.56** | A1477-a86 | **25.12** | **906.84** |
| C1277-a89 | **472.38** | A1502-a89 | **10023.45** | **5247.915** |

**Supplementary Table 3. Cytokines secreted by autologous naïve CD4+ CD45A+ T cells after 3, 5 and 7 days of co-culture with autonomously generated DCs from age-specific TCs stimulated with pediatric vaccines HBV, PCV and BCG.** BCG was used at 1:20v/v dilution; HBV and PCV were tested at 1:2v/v dilution. Supernatants were collected from several technical replicas per condition/time point. Values are the mean of 5 subject participants per age group (pg/mL). Background colors indicate statistically significant differences between particular vaccines and unstimulated (unstim) control (Paired observations). Number colors indicate statistically significant differences between particular conditions of Newborns and Adults (UnPaired observations). P=*<0.05; **<0.01; ***<0.001.

**Supplementary Table 4. Summary of *in vivo* infant T cell responses induced by autonomously generated DCs from human newborn tissue constructs immunized *in vitro* with pediatric vaccines BCG, HBV and PCV.** BCG *in vivo* data [3-22]; HBV *in vivo* data [23, 24]; and PCV *in vivo* data [25].

| **Vaccine** | **Doses** | **Significant responses** | ***In vitro*** | ***In vivo*** |
| --- | --- | --- | --- | --- |
| **BCG** | One at birth (BCG- or PPD-recall) | Induction of IL-1β, IL-2, IL-5, IL-6, IL-10, IL-12, IL-13, IL-17, TNF-α, IFN-γ | **✓** | **✓** |
|  | One at birth | Naïve CD4+ T cell proliferation | **✓** | **✓** |
|  | One at birth | Memory CD4+ T cell proliferation | **✓** | **✓** |
|  | One at birth | Increased plasma ADA-1 activity | **✓** | **✓** |
|  | One at birth | Strong Ag85A-specific CD4+ T cell proliferation | **✓** | **✓** |
| **HBV** | One at birth | No significant induction of IL-5, IL-13 and IFN-γ | **✓** | **✓** |
|  | Three dose series (HBsAg-recall) | Induction of IL-5 and IFN-γ | **(?)** | **✓** |
|  | One at birth | CD4+ T cell proliferation | **✓** | **(?)** |
|  | One at birth | Weak HBsAg-specific CD4+ T cell proliferation | **✓** | **✓** |
|  | One at Birth + infant Boost | Strong HBsAg-specific CD4+ T cell proliferation | **✓** | **✓** |
| **PCV** | One at birth | Induction of IL-1β, IL-6, IL-10, IL-12p70 and IFN-γ | **✓** | **(?)** |
|  | Three dose series (CRM197-recall) | Induction of IL-1β, IL-6, IL-10, IL-12p70 and IFN-γ | **(?)** | **✓** |
|  | One at birth | CD4+ T cell proliferation | **✓** | **(?)** |

## Supplementary Figures

**Supplementary Figure 1. Inter-assay reproducibility of autologous HBsAg-specific recall-responses from HBV *in vitro* immunization of ATCs.** Inter-assay reproducibility was retrospectively investigated after realizing 3 adult participants were unintentionally re-used for antigen specific challenge proliferation from HBV *in vitro* immunization. Blood donations occurred with > 1 year span in between them and were used in two separate occasions for same proliferation assays. DCs from HBV-stimulated ATCs (1:100v/v) were co-cultured with autologous CD4+ T cells for 7 days and then re-stimulated (boost in 100% autologous plasma) with a second round of autologous DCs from HBV-stimulated ATCs. At day 21 of culture, resting T cells were collected, counted and split to undergo antigen challenge for another 10 days using autologous monocytes and peptide pools as antigens. HBsAg = antigen-specific peptide pool. Scrambled = unspecific background peptide pool. Lymphoproliferation in response to peptide pools (Counts Per Minute or CPM) was measured at day 31. Three adult donors had their HBsAg-Abs titers checked each time they donated by The Department of Laboratory Medicine of Boston Children’s Hospital (mIU/mL). Mean proliferation levels and anti-HBsAg titers between independent assays performed almost a year apart with same donors show similar trends and no significant differences (Paired t-Test, N= 3).

**Supplementary Figure 2. Age-specific maturation of autonomously generated DCs after adjuvant and plasma type stimulation of TCs.** Changes on the surface Mean Fluorescent Intensities (MFI) of markers **a)** HLA-DR, **b)** CD86 and **c)** CD197 were measured by Flow Cytometry on DCs from age-specific TCs stimulated by relatively weak neonatal adjuvant Pam_3_CSK_4_ (TLR2/1A; Pam3, 10µg/mL) or the robust adjuvant resiquimod (TLR7/8A; R848, 50µM). To investigate the contribution of autologous plasma on DC maturation each experiment included conditions in which autologous plasma from the newborn and the adult were swapped, as described in Methods. Fetal Bovine Serum (FBS) was only tested for Pam3. Ø = unstimulated TCs. Black stars compared every condition to unstimulated autologous plasma controls, per age group; red stars compared every condition to unstimulated control (Ø), per plasma type group; blue stars compared the effect of plasma type on each condition, per age group. N = 3-10 donors per age group with >7 technical replicas per condition. P=*<0.05; **<0.01; ***<0.001.

**Supplementary Figure 3. Uptake and processing of *mycobacterium bovis* by DCs derived from BCG *in vitro* immunized age-specific TCs.** BCG vaccine, consisting of live attenuated *Mycobacterium bovis* bacteria, was labeled with blue-fluorescent DraQ5 dye for DNA and Orange-red fluorescent lipophilic dye 1,1'-Dioctadecyl-3,3,3',3'-Tetramethylindocarbocyanine Perchlorate for bacteria lipids [26]. Age-specific TCs were cultured with labeled BCG vaccine (1:10v/v, 20,000-80,000 colony forming units [27] in autologous plasma for 48h. Reverse transmigrated DCs were carefully harvested and stained with mouse anti-human HLA-DR-FITC (HLA-DR, green), as described in Methods. Representative 0.25µm thick optic focal plane cuts inside cells shows bacterial lipids (red) in vesicles. White star box shows red-labeled BCG vaccine suspension.

**Supplementary Figure 4. Autologous naïve T cell proliferation induced by DCs autonomously generated from age-specific TCs stimulated with BCG.** Proliferation of Naïve CD4+ CD45RA+ T cells (Counts Per Minute or CPM) was assessed at days 3, 5 and 7. BCG was used at 1:20v/v dilution (squares). Red dotted line indicates background proliferation by unstimulated controls (Ø, circles), naïve T cells alone (no DCs, cones), naïve T cells plus live attenuated BCG (no DCs, rhombus) and BCG vaccine alone with no antibiotics (triangles). N= 5-7 participants/age group, per condition. Black stars compared BCG vs. unstimulated controls (Ø) on same age group. P=*<0.05; **<0.01; ***<0.001.

**Supplementary Figure 5. ADA-1 activity (U/L) by non-heated plasmas from newborns (N=10) and adults (N=7).** U/L = Units per liter. P= *<0.05; **<0.01; ***<0.001.


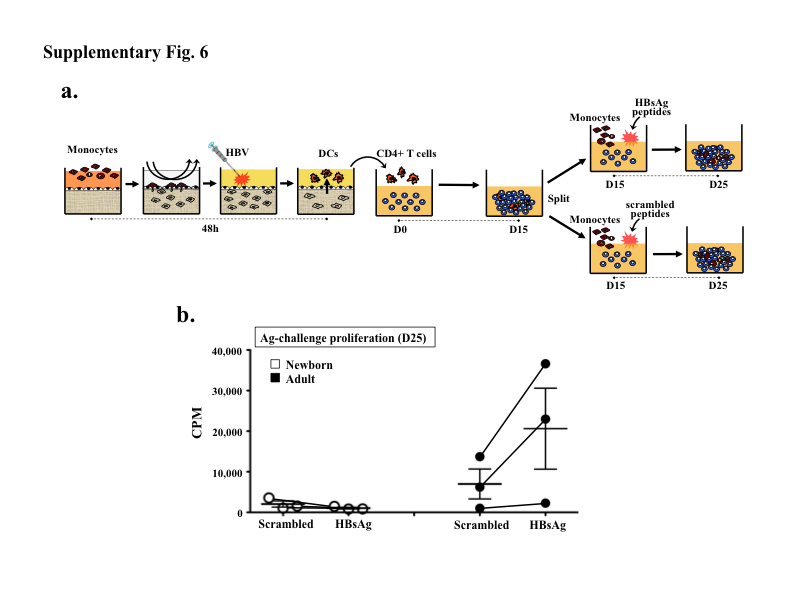


**Supplementary Figure 6. A single dose HBV *in vitro* immunization is insufficient to detect autologous newborn HBsAg-specific recall-proliferation. a.** Assay design to assess autologous newborn HBsAg-specific recall-proliferation after a single dose of HBV *in vitro*. DCs from age-specific TCs stimulated with HBV (1:100v/v) were co-cultured with autologous CD4+ T cells for 15 days (D15) and split (5,000 cells/well) to undergo antigen challenge (Ag-challenge) for 10 more days (D25) using autologous monocytes with either a peptide pool of antigen-specific Hepatitis B virus Surface Antigen (HBsAg) or scrambled sequences control (**Supplementary Table 1**). Only 100% autologous plasma was used. **b.** Ag-challenge proliferation was assessed at day 25 by H^3^-thymidine incorporation method (CPM= Counts Per Minute), as described in methods. Each data point reflects the mean of at least seven technical replicas per study participant. Connecting lines indicate same study subject (N= 3).


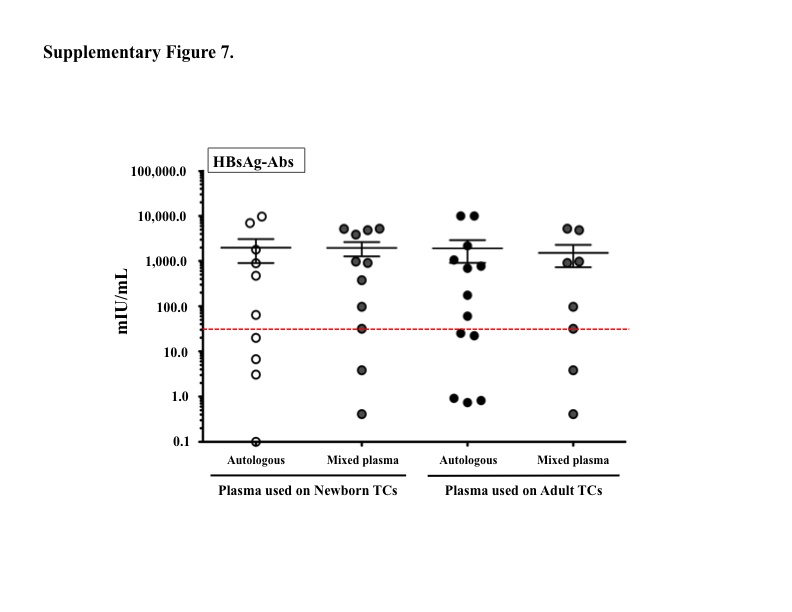


**Supplementary Figure 7. Mean antibody titers against Hepatitis B surface Antigen (HBsAg-Abs) found in plasmas used for HBV antigen challenge studies.** HBsAg is a surface protein of Hepatitis B virus and the only targeted antigen in HBV vaccine. Red dotted line indicates HBsAg-Abs titers above the correlate of protection of ≥12.00 mIU/mL recommended by The Department of Laboratory Medicine of Boston Children’s Hospital, where these titers were independently measured (**Supplementary Table 2**). Titers of mixed plasmas were not measured but inferred from mixing 1:1v/v autologous plasmas from one newborn and one adult, simultaneously being tested on that same assay. mIU/mL = mili-International Units per mL.


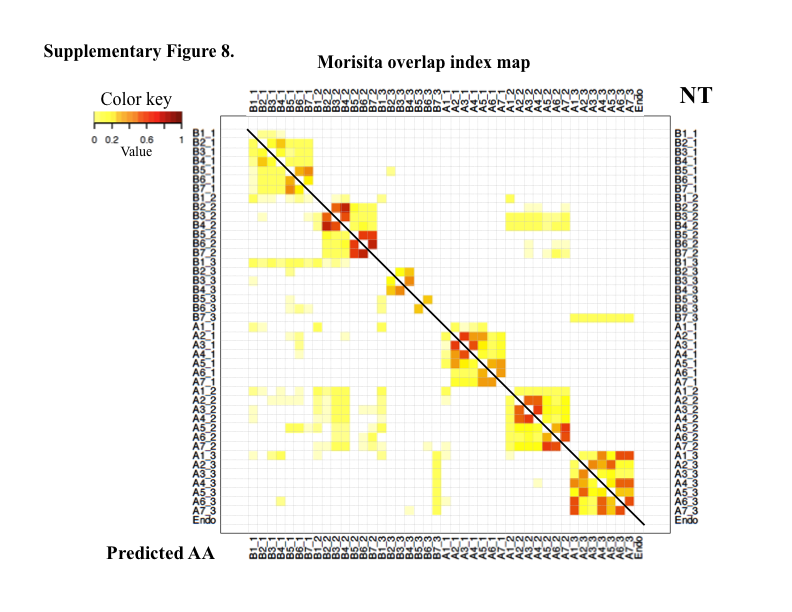


**Supplementary Figure 8. Morisita Overlap Index map of all productive rearranged TCR sequences found on three assays to assess autologous antigen-specific recall-responses after HBV newborn *in vitro* immunization.** Autologous CD4+ T cells (day 0) were co-cultured with for 7 days with DCs from age-specific TCs stimulated with HBV (1:100v/v) and then re-stimulated with a second round of autologous DCs form HBV-stimulated TCs. At day 21 of further culture, resting T cells were collected (samples and ), counted and split (5,000 cells/well) to undergo antigen challenge (recall-responses) for another 10 days using autologous monocytes and a peptide pool approach: antigen-specific HBsAg ( and ) vs. background control scrambled sequences ( and ). From day 7 onward, half the experiments continue their culture under either 100% autologous plasma (, and ) or a 1:1v/v mix of newborn and adult plasmas (, and ). Cell samples from steps , , , , , , and according to assay design (**Fig. 13**) were taken for DNA extraction and high-throughput sequencing of the TCRβ CDR3 region, as described in Methods (N=3/age-group). TCR sequencing generated 164,738 productive rearranged TCRs from 42 test samples (6 study participants) and one extra sample of single donor endothelial cells (same HUVECs used for all experiments), sequenced as internal control (Endo). A Morisita Overlap Index map was generated as a statistical measure of dispersion to compare TCR overlap between participants and conditions (ImmunoSEQ® Analyzer, Adaptive Biotechnologies; Seattle, WA). The graphic map depicts and compares detected nucleotides (NT - top) and predicted aminoacid (AA - bottom) sequences. Samples are identified combining letters and numbers. Letter B is for “Newborn babies” and letter A is for “Adults”; following numbers 1 to 7 are the samples (, , , , , , and ); last number identifies the study participant (e.g. Baby 1, Adult 3). Example: B5_1 = test condition of newborn participant #1. Color key indicates the level of exact-matching overlapping between the sequences of all samples. Both the nucleotide (NT) and predicted aminoacid (AA) sequences indicated an acceptable level of independency (TCR clustering) between the six non-consanguineous participants. Moreover, no overlap was noted between the six samples and endothelium control.

##
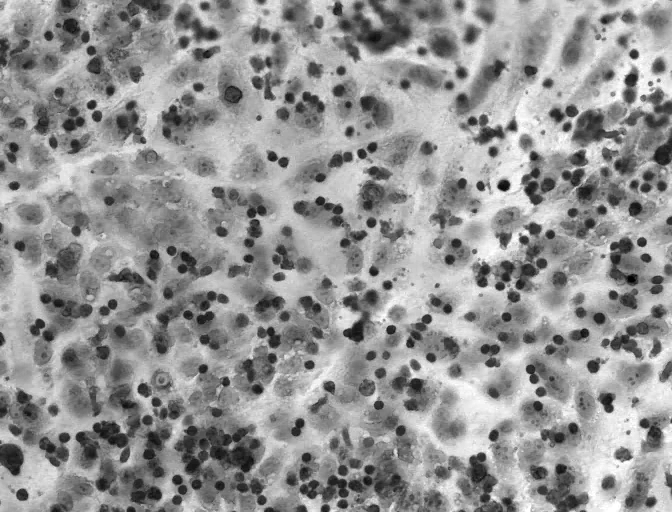
Supplementary Videos

**Supplementary Video S1.** **Trans-perpendicular video of a Tissue Construct after 48h of culture.** Video was generated using a series of pictures taken top to the bottom at approximately every 20µm. Reverse transmigrated cells were removed from the luminal side of the endothelium before, fixing and staining with Haematoxylin-Eosin.

**
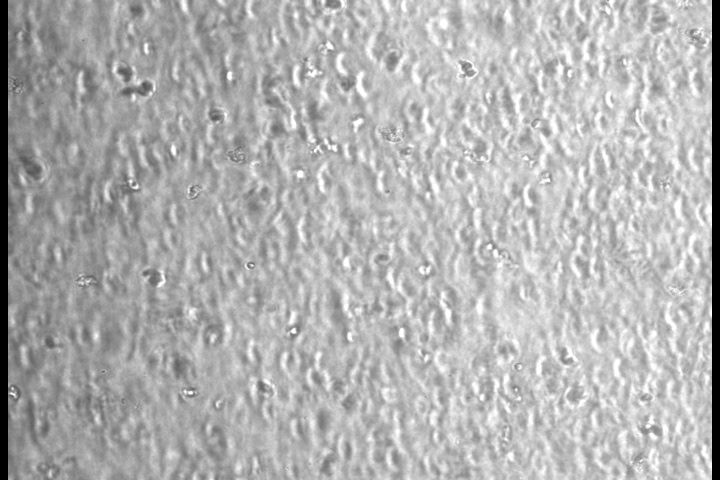
**

**Supplementary Video S2.** **Time-lapse video of live monocytes at ~50µm inside of a Tissue Construct after 18h of culture.** Video was generated with pictures taken with an inverted microscope every 30 seconds for a time span of about 15 minutes and compressed to ~8 seconds.

**
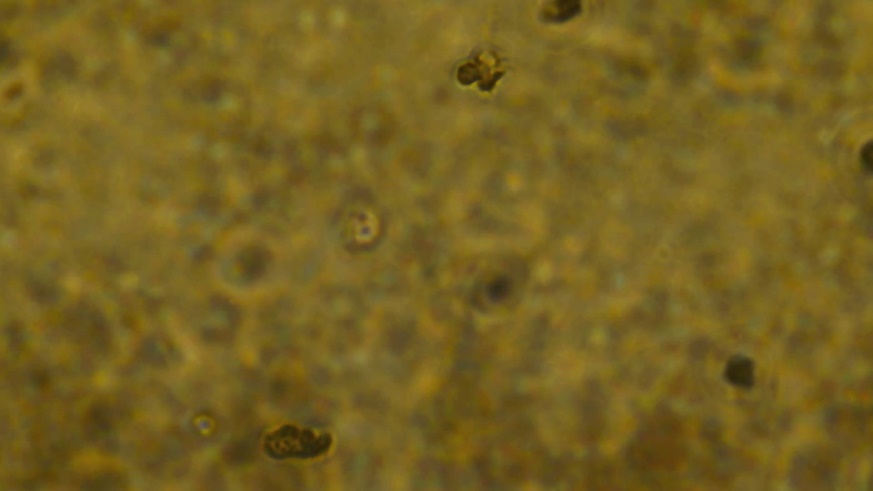
**

**Supplementary Video S3. Time-lapse video of live Dendritic Cells projecting dendrites (sampling) at ~50µm inside a Tissue Construct after 24h of culture.** Video was made with pictures taken with an inverted microscope every ~15 seconds for a time span of about 10 minutes and compressed to ~15 seconds.

**
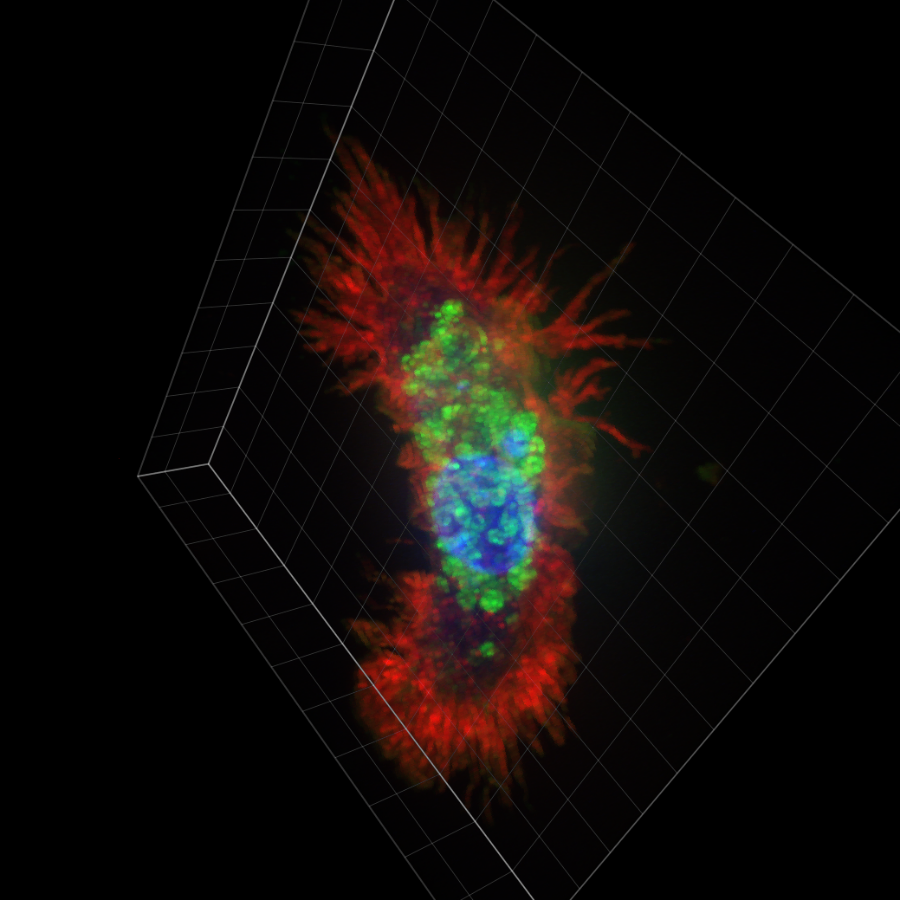
**

**Supplementary Video S4. Three-dimensional rotational-axe Confocal video-microscopy reconstruction (Slidebook® software) of an adult immature Dendritic Cell derived from an unstimulated Tissue Construct.** Cytoplasmic stored HLA-DR class II molecules are seen in green (inside the cell), F-actin filaments (cytoskeleton) are seen in red and DNA is seen in blue.

**
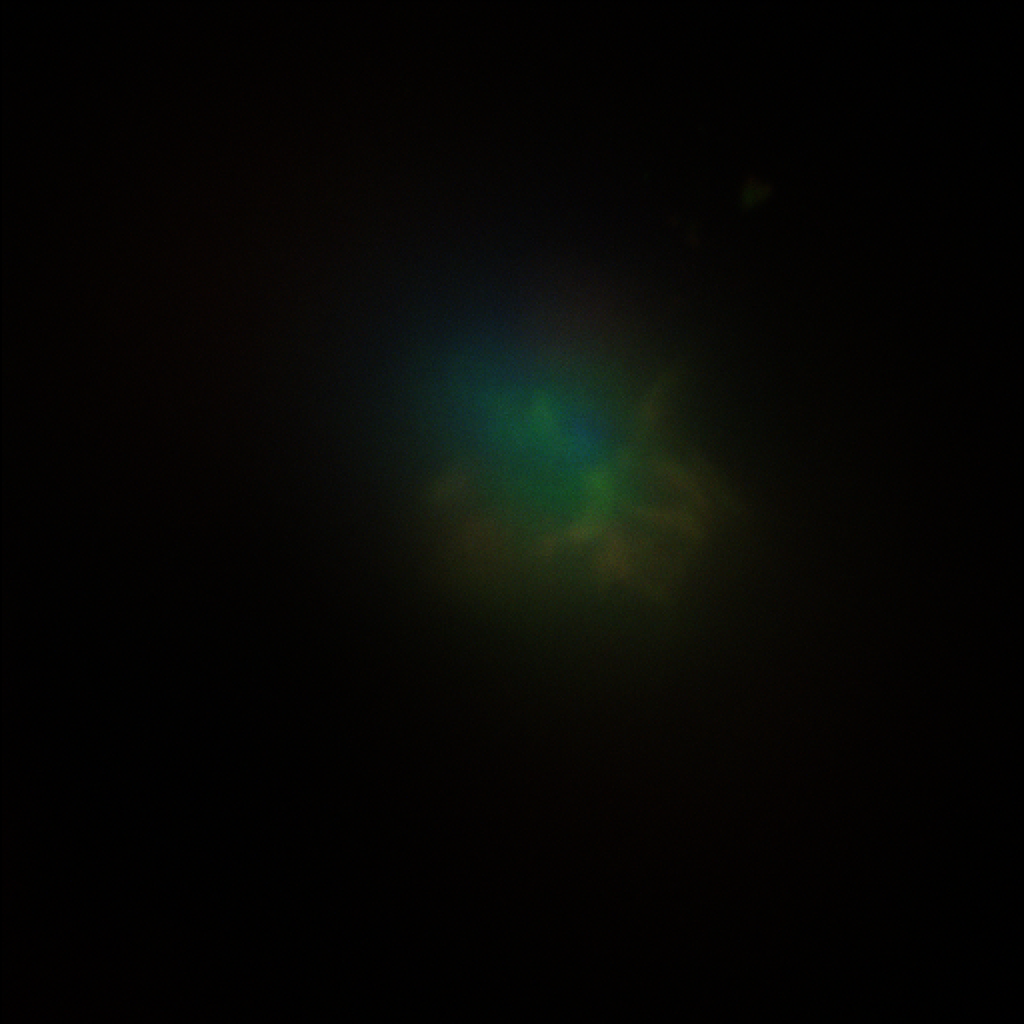
**

**Supplementary Video S5.** **Plane-by-plane video-microscopy reconstruction of an adult immature Dendritic Cell (unstimulated Tissue Construct).** Confocal images were taken 0.2µm apart. Cytoplasmic stored HLA-DR class II molecules are seen in green (inside the cell), F-actin filaments (cytoskeleton) are seen in red and DNA is seen in blue.

**
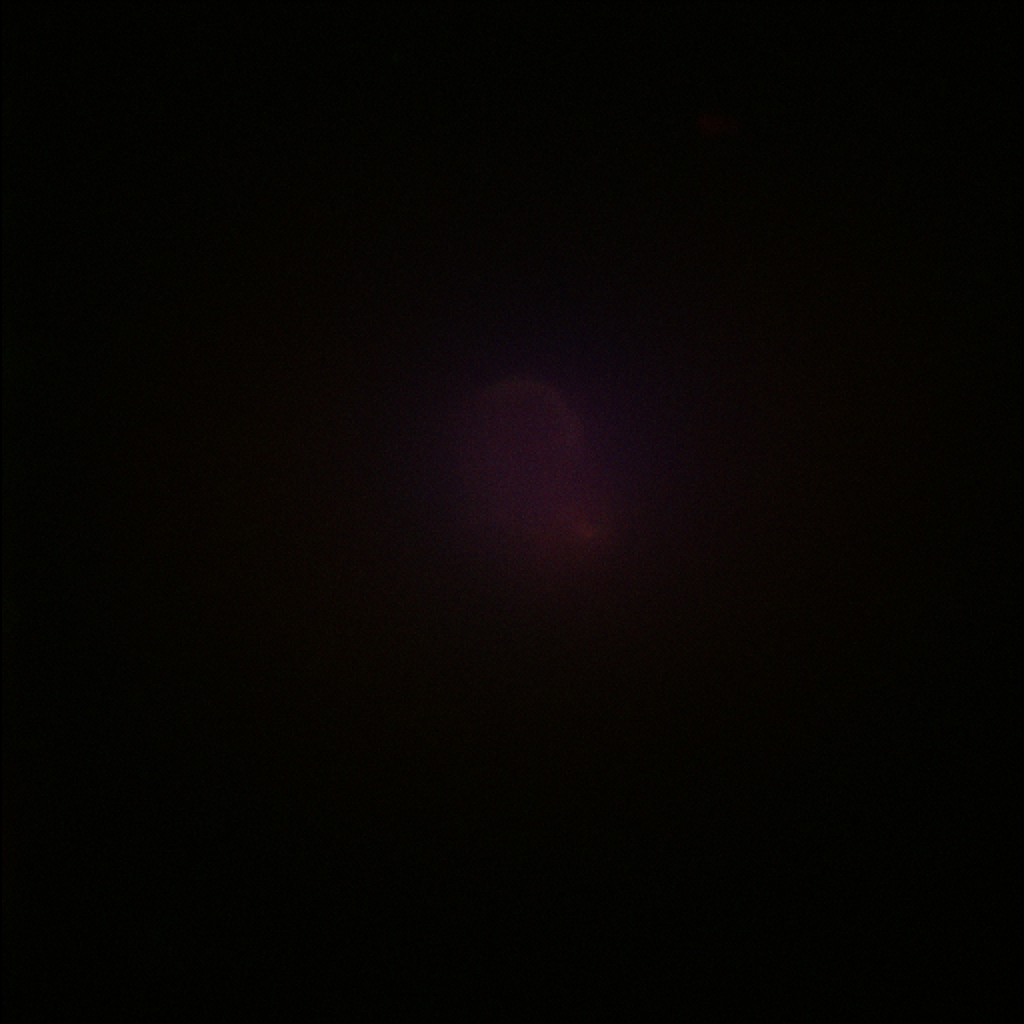
**

**Supplementary Video S6.** **Plane-by-plane video-microscopy reconstruction of a newborn immature Dendritic Cell (unstimulated Tissue Construct).** Confocal images were taken 0.2µm apart. Cytoplasmic stored HLA-DR class II molecules are seen in green (inside the cell), F-actin filaments (cytoskeleton) are seen in red and DNA is seen in blue.

1. Huygen, K., et al., *Mapping of TH1 helper T-cell epitopes on major secreted mycobacterial antigen 85A in mice infected with live Mycobacterium bovis BCG.* Infect Immun, 1994. **62**(2): p. 363-70.

2. Desombere, I., et al., *Characterization of the T cell recognition of hepatitis B surface antigen (HBsAg) by good and poor responders to hepatitis B vaccines.* Clin Exp Immunol, 2000. **122**(3): p. 390-9.

3. Jensen, K.J., et al., *Heterologous immunological effects of early BCG vaccination in low-birth-weight infants in Guinea-Bissau: a randomized-controlled trial.* J Infect Dis, 2015. **211**(6): p. 956-67.

4. Casanova, J.L. and L. Abel, *Genetic dissection of immunity to mycobacteria: the human model.* Annu Rev Immunol, 2002. **20**: p. 581-620.

5. Pachlopnik Schmid, J., T. Gungor, and R. Seger, *Modern management of primary T-cell immunodeficiencies.* Pediatr Allergy Immunol, 2014. **25**(4): p. 300-13.

6. Murray, R.A., et al., *Bacillus Calmette Guerin vaccination of human newborns induces a specific, functional CD8+ T cell response.* J Immunol, 2006. **177**(8): p. 5647-51.

7. Randhawa, A.K., et al., *Association of human TLR1 and TLR6 deficiency with altered immune responses to BCG vaccination in South African infants.* PLoS Pathog, 2011. **7**(8): p. e1002174.

8. Djuardi, Y., et al., *A longitudinal study of BCG vaccination in early childhood: the development of innate and adaptive immune responses.* PLoS One, 2010. **5**(11): p. e14066.

9. Sartono, E., et al., *Oral polio vaccine influences the immune response to BCG vaccination. A natural experiment.* PLoS One, 2010. **5**(5): p. e10328.

10. Burl, S., et al., *Delaying bacillus Calmette-Guerin vaccination from birth to 4 1/2 months of age reduces postvaccination Th1 and IL-17 responses but leads to comparable mycobacterial responses at 9 months of age.* J Immunol, 2010. **185**(4): p. 2620-8.

11. Akkoc, T., et al., *Neonatal BCG vaccination induces IL-10 production by CD4+ CD25+ T cells.* Pediatr Allergy Immunol, 2010. **21**(7): p. 1059-63.

12. Kagina, B.M., et al., *Delaying BCG vaccination from birth to 10 weeks of age may result in an enhanced memory CD4 T cell response.* Vaccine, 2009. **27**(40): p. 5488-95.

13. Finan, C., et al., *Natural variation in immune responses to neonatal Mycobacterium bovis Bacillus Calmette-Guerin (BCG) Vaccination in a Cohort of Gambian infants.* PLoS One, 2008. **3**(10): p. e3485.

14. Matee, M., et al., *Baseline mycobacterial immune responses in HIV-infected adults primed with bacille Calmette-Guerin during childhood and entering a tuberculosis booster vaccine trial.* J Infect Dis, 2007. **195**(1): p. 118-23.

15. Watkins, M.L., et al., *Exposure of cord blood to Mycobacterium bovis BCG induces an innate response but not a T-cell cytokine response.* Clin Vaccine Immunol, 2008. **15**(11): p. 1666-73.

16. Vekemans, J., et al., *Neonatal bacillus Calmette-Guerin vaccination induces adult-like IFN-gamma production by CD4+ T lymphocytes.* Eur J Immunol, 2001. **31**(5): p. 1531-5.

17. Soares, A.P., et al., *Bacillus Calmette-Guerin vaccination of human newborns induces T cells with complex cytokine and phenotypic profiles.* J Immunol, 2008. **180**(5): p. 3569-77.

18. Marchant, A., et al., *Newborns develop a Th1-type immune response to Mycobacterium bovis bacillus Calmette-Guerin vaccination.* J Immunol, 1999. **163**(4): p. 2249-55.

19. Jones, B.E., et al., *Relationship of the manifestations of tuberculosis to CD4 cell counts in patients with human immunodeficiency virus infection.* Am Rev Respir Dis, 1993. **148**(5): p. 1292-7.

20. Oni, T., et al., *Smoking, BCG and employment and the risk of tuberculosis infection in HIV-infected persons in South Africa.* PLoS One, 2012. **7**(10): p. e47072.

21. Oni, T., et al., *Risk factors associated with indeterminate gamma interferon responses in the assessment of latent tuberculosis infection in a high-incidence environment.* Clin Vaccine Immunol, 2012. **19**(8): p. 1243-7.

22. Arikan, C., et al., *Bacillus Calmette-Guerin-induced interleukin-12 did not additionally improve clinical and immunologic parameters in asthmatic children treated with sublingual immunotherapy.* Clin Exp Allergy, 2004. **34**(3): p. 398-405.

23. Ota, M.O., et al., *Hepatitis B immunisation induces higher antibody and memory Th2 responses in new-borns than in adults.* Vaccine, 2004. **22**(3-4): p. 511-9.

24. Avanzini, M.A., et al., *Increment of recombinant hepatitis B surface antigen-specific T-cell precursors after revaccination of slow responder children.* Vaccine, 2001. **19**(20-22): p. 2819-24.

25. van den Biggelaar, A.H., et al., *Pneumococcal conjugate vaccination at birth in a high-risk setting: no evidence for neonatal T-cell tolerance.* Vaccine, 2011. **29**(33): p. 5414-20.

26. Kumari, M. and R.K. Saxena, *Relative efficacy of uptake and presentation of Mycobacterium bovis BCG antigens by type I mouse lung epithelial cells and peritoneal macrophages.* Infect Immun, 2011. **79**(8): p. 3159-67.

27. Institut, S.S., *Description of BCG VACCINE SSI.* 2011.
